# Promotion of stem cell-like phenotype of lung adenocarcinoma by FAM83A via stabilization of ErbB2

**DOI:** 10.1038/s41419-024-06853-w

**Published:** 2024-06-28

**Authors:** Ye Yuan, Liang Hao, Jing-Shan Huang, Fu-Ying Zhao, Ying-Hua Ju, Jia-Mei Wang, Ting Zhang, Bai-Qiang Li, Zhan-Wu Yu

**Affiliations:** 1 Central Laboratory, Cancer Hospital of China Medical University, Liaoning Cancer Hospital & Institute, Cancer Hospital of Dalian University of Technology, Shenyang, 110042 China; 2 Department of Biochemistry & Molecular Biology, China Medical University, Shenyang, 110026 China; 3 Department of Chemistry, School of Forensic Medicine, China Medical University, Shenyang, 110026 China; 4 Department of Thoracic Surgery, the Shengjing Hospital, China Medical University, Shenyang, 110001 China; 5 Department of Laboratory Medicine, the 1st affiliated hospital, China Medical University, Shenyang, 110001 China; 6 Department of Thoracic Surgery, Cancer Hospital of China Medical University, Liaoning Cancer Hospital & Institute, Cancer Hospital of Dalian University of Technology, Shenyang, 110042 China

**Keywords:** Prognostic markers, Cancer stem cells

## Abstract

Lung cancer stands as the leading cause of mortality among all types of tumors, with over 40% of cases being lung adenocarcinoma (LUAD). Family with sequence similarity 83 member A (FAM83A) emerges as a notable focus due to its frequent overexpression in LUAD. Despite this, the precise role of FAM83A remains elusive. This study addresses this gap by unveiling the crucial involvement of FAM83A in maintaining the cancer stem cell-like (CSC-like) phenotype of LUAD. Through a global proteomics analysis, the study identifies human epidermal growth factor receptor 2 (HER2 or ErbB2) as a crucial target of FAM83A. Mechanistically, FAM83A facilitated ErbB2 expression at the posttranslational modification level via the E3 ubiquitin ligase STUB1 (STIP1-homologous U-Box containing protein 1). More importantly, the interaction between FAM83A and ErbB2 at Arg241 promotes calcineurin (CALN)-mediated dephosphorylation of ErbB2, followed by inhibition of STUB1-mediated ubiquitin-proteasomal ErbB2 degradation. The maintenance of the CSC-like phenotype by FAM83A, achieved through the posttranslational regulation of ErbB2, offers valuable insights for identifying potential therapeutic targets for LUAD.

## Introduction

Lung cancer ranks second among frequently diagnosed neoplastic disorders, surpassed only by breast cancer, yet it bears the highest mortality rates, contributing to 18% of total cancer deaths [1, 2]. Lung adenocarcinoma (LUAD), a subtype of non-small cell lung cancer (NSCLC), accounts for around 40% of all lung cancers [3–5]. A majority of patients may have metastases or be in an advanced state when diagnosed, limiting therapeutic options and resulting in a poor prognosis [6]. Despite advancements in therapeutic regimes for LUAD, clinical outcomes remain dismal, especially for patients who undergo recurrence and metastasis after treatment [6, 7]. Therefore, a comprehensive understanding of the underlying molecular mechanisms associated with LUAD may help explore effective targets against it and improve the clinical outcome of patients.

Cancer stem cells (CSCs) are a subset of cancer cells with enhanced capacities for self-renewal and differentiation into different cell lineages, believed to initiate tumor growth and promote cancer progression [8–10]. Although a large amount of patients have received clinical treatment, they still experience poor prognosis due to drug resistance, recurrence, or metastasis, which is attributed to the presence of CSCs. Compelling evidence has uncovered that tumor progression is intricately associated with the existence of CSCs within the tumor bulk, including LUAD, ovarian cancer, hematologic tumors, and so on [11, 12]. CSC-like phenotypes also play important roles in metastasis, recurrence, and other malignant phenotypes, which can be characterized by several methods including spheroid formative capacity, stem cell marker expression, tumorigenic potential, and increased invasiveness [12–14]. CSC-like phenotypes are regulated by the integrated transcriptional, post-transcriptional, metabolic, and epigenetic regulatory networks [15, 16]. Importantly, accumulating evidence has emerged that CSCs in LUAD are critical in tumorigenesis, metastasis, and recurrence [17]. Therefore, a comprehensive exploration of the biological characteristics and self-renewal mechanisms of CSCs will provide insights into the therapeutic strategies for the progression and recurrence of LUAD.

A family with sequence similarity 83 member A (FAM83A), also known as tumor-specific antigen-9 (BJ-TSA-9), is located on chromosome 8q24 [18]. As the smallest member of the FAM83 family (FAM83A to H), FAM83A comprises 434 amino acids and contains the DUF1669 domain, serine-rich domains, and proline-rich domains (PRDs) [19]. Notably, the conserved DUF1669 domain at its N-terminus was thought to be involved in tumor progression, leading to FAM83A being initially identified as a potential cancer biomarker in 2005 [19, 20]. Some previous studies have reported that the elevated expression of FAM83A in a substantial fraction of cancers, including breast cancer, NSCLC [21, 22], cervical cancer [23], hepatocellular carcinoma [24], and pancreatic ductal adenocarcinoma [25], indicating abnormal upregulation of FAM83A may indeed promote tumorigenesis and cancer progression [20]. Increasing evidence shows that FAM83A plays its pro-tumoral role related to some classic tumor-related signaling pathways, such as the PI3K/AKT pathway, MAPK pathway, and Wnt/β-catenin signaling pathway [22, 24, 25]. However, the detailed molecular mechanisms of FAM83A in LUAD remain to be thoroughly investigated.

In this study, we figured out that FAM83A was upregulated in LUAD and required for the maintenance of CSC-like phenotypes. Mechanically, our investigation delved into the posttranslational regulation of human epidermal growth factor receptor 2 (ErbB2, encoding HER2) by FAM83A. Specifically, we identified that the interaction between FAM83A and ErbB2 at Arg241 promoted the dephosphorylation of ErbB2 by calcineurin (CALN), thereby significantly suppressing its ubiquitination mediated by STIP1-homologous U-Box containing protein 1 (STUB1). The present findings might provide new insights into the specific role of FAM83A in LUAD progression.

## Results

### FAM83A expression was abnormally upregulated in LUAD tissues

**Fig. 1:**
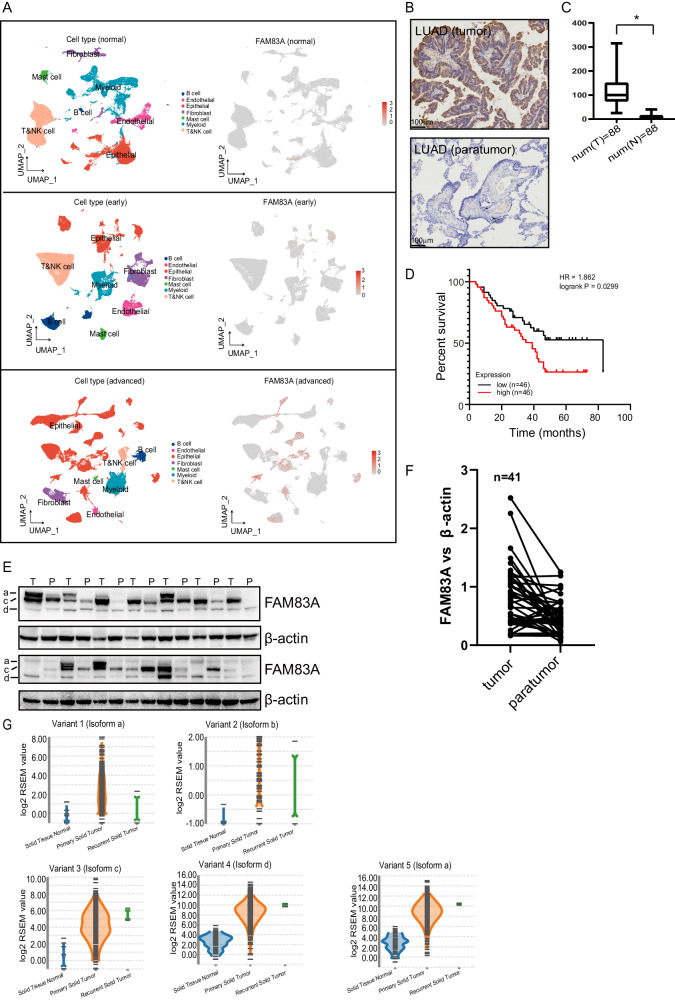
FAM83A expression was abnormally upregulated in LUAD tissues. **A** UMAP view of total cells obtained from scRNA sequencing samples, color-coded by assigned cell type, with marked FAM83A expression (Top: normal, middle: early stage, bottom: advanced stage). **B** Representative IHC staining of FAM83A in 92 LUAD tissues, where cytoplasm-stained FAM83A was expressed in the tumor but not in the paratumor tissues. **C** Box plots showing the expression of FAM83A according to IHC scores in 88 LUAD tumor and paratumor tissues. **D** Kaplan–Meier plot analyzing the overall survival of LUAD patients stratified by high or low FAM83A IHC intensity. **E** Western blot analysis of FAM83A protein levels in paired fresh LUAD (T) and peripheral normal (P) tissues (*n* = 41), the representative images were shown. **F** Scatter plots showing the relative expression of FAM83A in paired LUAD (T) and peripheral normal (P) tissues. **G** Violin plots showing the expression of different FAM83A variants in tumor specimens relative to peritumor by analyzing TSVdb online. Data represent means ± SD of three independent experiments; *P < 0.05; ns not significant.

To investigate the potential significance of FAM83A in the progression of LUAD, we analyzed single-cell RNA sequencing (scRNA-seq) data GSE119911 from the Gene Expression Omnibus (GEO). Our findings revealed high expression of FAM83A in the epithelial cells of advanced tumor (Fig. 1A). Subsequently, immunohistochemical (IHC) analysis of 92 LUAD patients confirmed that FAM83A expression was significantly increased in most tumor specimens relative to peritumor tissues (Fig. 1B, C). The clinical characteristics of LUAD patients with low FAM83A expression and high FAM83A expression are shown in Table 1. Importantly, the Cox proportional hazards model identified high FAM83A expression as an independent prognostic factor with respect to overall survival (Fig. 1D). Further validation through immunoblot analysis of lysates obtained from surgical samples of LUAD patients confirmed the increase of FAM83A expression in most tumors compared with corresponding peritumor tissues. Multiple bands were observed in most tissues (Fig. 1E, F). Investigation into alternative splicing of FAM83A revealed five variants, encoding four isoforms. Analyzing TSVdb online data confirmed the expression of all five variants in tumor specimens relative to peritumor tissues, with variants 3, 4, and 5, which encode isoforms c, d, and a, respectively, showing abundant expression (Fig. 1G).

**Table 1:** FAM83A expression with clinical characteristics in 92 LUAD samples.

Clinical characteristics	FAM83A expression		*P* value
	low	High	
Age			0.834
≤63 (Median)	25	24	
>63	21	22	
Gender			0.834
Male	25	26	
Female	21	20	
Stage			0.053
I&II	33	24	
III&IV	13	22	
T Classification			0.005
T1&T2	42	31	
T3&T4	4	15	
N Classification			0.002
N0	21	13	
N1	21	13	
N2	4	19	
N3	0	1	
M Classification			1.000
M0	45	45	
M1	1	1	
Survival status			0.002
Survival	28	13	
Death	18	33	

### CSC-like phenotypes of LUAD cells were suppressed by FAM83A knockdown

**Fig. 2:**
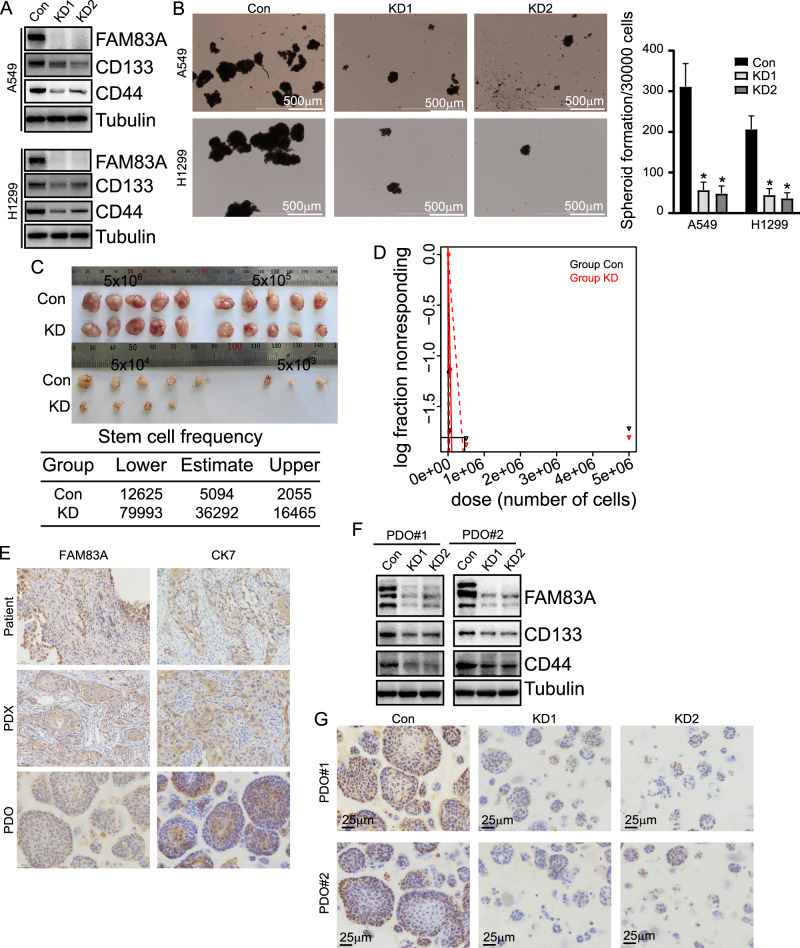
CSC-like phenotypes of LUAD cells were suppressed by FAM83A knockdown. **A** A549 and H1299 cells were infected with sgRNA-guided FAM83A using the CRISPR/Cas9 system. Western blot was performed using the indicated antibodies. **B** Representative photographs of spheroids derived from control or FAM83A KD A549 and H1299 cells and their quantification. **C** Indicated control or FAM83A knockdown A549 cells with serial dilutions were injected intracutaneously into nude mice. Tumors were excised after the mice were sacrificed on day 28 (*n* = 5 mice/group). **D** The frequency of CSCs was predicted using ELDA, and a log-fraction plot of the limiting dilution model was extracted. The slope of the line is the log-active cell fraction, and the dotted lines denote the 95% confidence interval. **E** Representative IHC staining with FAM83A in PDXs and PDOs of LUAD. Cytokeratin 7 (CK7) was used as the LUAD marker. **F** PDOs of LUAD were infected with gRNA-guided FAM83A using the CRISPR/Cas9 system. Western blot was performed using the indicated antibodies. **G** Representative IHC staining with control or FAM83A knockdown in PDOs of LUAD. Data represent means ± SD of three independent experiments; *P < 0.05; ns not significant.

To confirm the potential role of FAM83A in LUAD, we employed the CRISPR/Cas9 system to downregulate FAM83A in A549 and H1299 LUAD cells (Fig. 2A). Western blot demonstrated that FAM83A knockdown decreased the expression of the CSC markers CD133 and CD44 in LUAD compared with the control cells. Moreover, FAM83A knockdown significantly suppressed the spheroid formative capacity (Fig. 2B). Additionally, the formation of xenografted tumors by A549 cells was markedly hindered by FAM83A knockdown in nude mice (Fig. 2C). The CSC-like phenotypes of cells were then evaluated using an online tool for extreme limiting dilution analysis (http://bioinf.wehi.edu.au/software/elda/). The lower frequency of repopulating for control A549 cells (1/12625) was even larger than the upper frequency of FAM83A knockdown cells (1/16465), indicating CSC-like phenotypes were significantly decreased by FAM83A knockdown (Fig. 2D). We further established patient-derived xenografts (PDXs) and patient-derived organoids (PDOs) using LUAD tissues, confirming their high expression of FAM83A by IHC (Fig. 2E). Western blot demonstrated that FAM83A knockdown decreased the expression of CD133 and CD44 in PDOs (Fig. 2F). Furthermore, the sizes of PDOs were significantly reduced by FAM83A knockdown (Fig. 2G).

### Multiomics showed a positive correlation between FAM83A and ErbB2 expression in LUAD

**Fig. 3:**
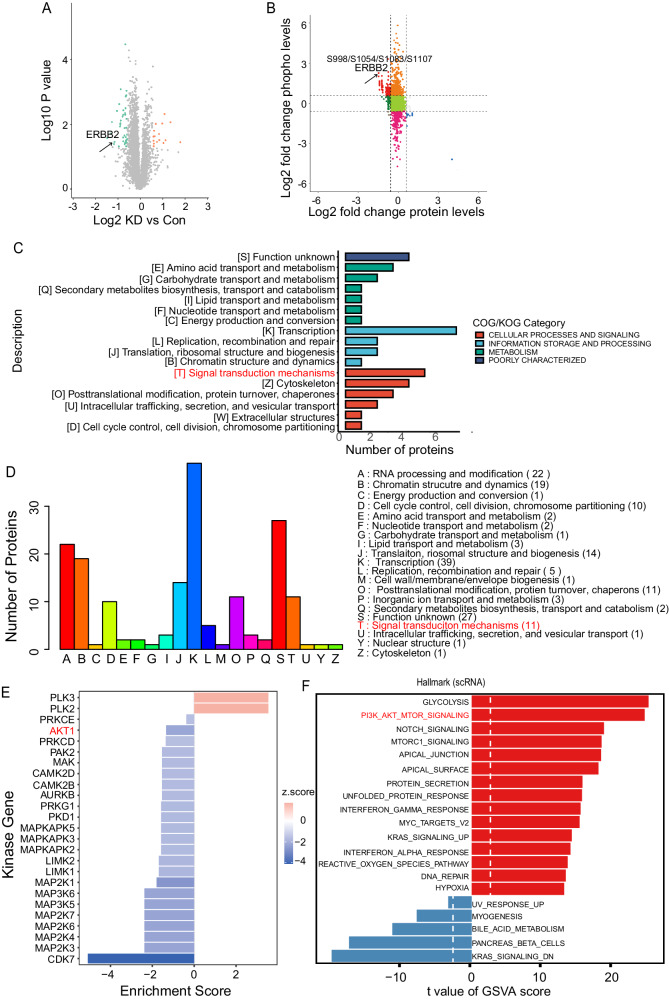
Multiomics showed a positive correlation between FAM83A and ErbB2 expression in LUAD. **A** The volcano plot of variable expression genes in LUAD cells between control and FAM83A knockdown by proteomics. Upregulated genes (FC >2) are colored in red while downregulated genes (FC less than −2) are colored in blue. **B** The volcano plot of variable expression genes in LUAD cells between control and FAM83A knockdown by proteomics and phosphorylation proteomics. **C** COG analysis of protein proteomics data of LUAD cells with control and FAM83A knockdown. **D** COG analysis of phosphorylation proteomics data of LUAD cells with control and FAM83A knockdown. **E** KSEA analysis of kinase activity data of LUAD cells with control and FAM83A knockdown. **F** GSVA analysis of single-cell RNA sequencing of 41 LUAD tissues. Data represent means ± SD of three independent experiments; *P < 0.05; ns not significant.

To explore the possible mechanisms implicated in the promotion of the CSC-like phenotypes by FAM83A, we conducted proteomics, which identified ErbB2 as a significantly downregulated target upon FAM83A knockdown (Fig. 3A). Furthermore, phosphorylation proteomics identified significant upregulation of ErbB2 phosphorylation in FAM83A knockdown cells (Fig. 3B). Clusters of orthologous groups (COG) analysis demonstrated that the proteomics and kinomics regulated by FAM83A were involved in classic oncogenic signal transduction pathways (Fig. 3C, D). Kinase substrate enrichment analysis (KSEA) recapitulated the suppression of AKT1, which is related to ErbB2, following FAM83A knockdown (Fig. 3E). Gene set variation analysis (GSVA) of scRNA-seq data also confirmed a high correlation between FAM83A expression and the activation of the PI3K-AKT signaling pathway in LUAD tissues (Fig. 3F).

### FAM83A knockdown decreased the stability of ErbB2

**Fig. 4:**
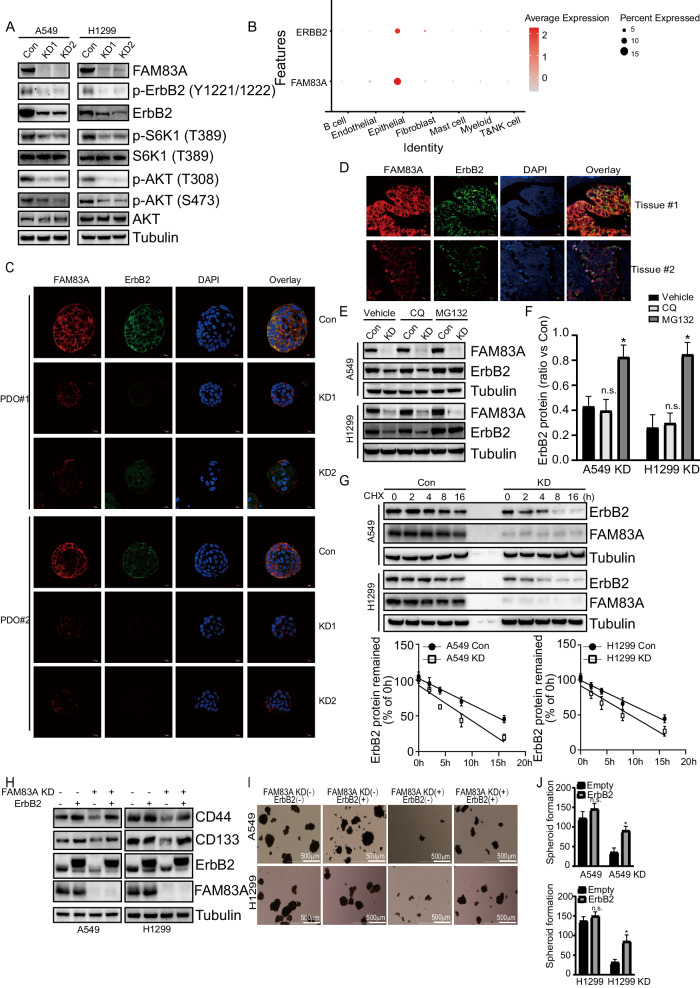
FAM83A knockdown decreased stability of ErbB2. **A** Western blot analysis of protein expression in A549 and H1299 cells with control or FAM83A knockdown, using the indicated antibodies. **B** Bubble plots showing the positive correlation between FAM83A and ErbB2 expression in epithelial tumor cells by scRNA-seq data analysis. **C** Immunofluorescence analysis of ErbB2 expression in PDO samples with control or FAM83A knockdown. **D** Immunofluorescence analysis of FAM83A and ErbB2 expression in LUAD tissues. **E**, **F** Western blot analysis of indicated protein expression levels in control or FAM83A knockdown cells treated with MG132 or CQ. In FAM83A knockdown cells, ErbB2 band intensities were measured, normalized by Tubulin, and the protein levels relative to those of vehicle cells are noted. **G** Western blot analysis of ErbB2 stability in Control or FAM83A knockdown cells treated with cycloheximide (CHX) for the indicated times. Representative images were provided. The band intensity was measured, normalized by Tubulin, and ErbB2 remaining relative to those of 0 h are plotted. **H** Western blot analysis of control or FAM83A knockdown cells with overexpressing ErbB2. The analysis focused on the indicated protein expression levels. **I**, **J** Representative photographs of spheroids derived from above LUAD cells and their numbers. Data represent means ± SD of three independent experiments; *P < 0.05; ns not significant.

The downregulation of ErbB2 and the activity of its related AKT signaling pathway by FAM83A knockdown was confirmed in LUAD cells (Fig. 4A). In addition, a significant co-expression of FAM83A and ErbB2 was observed in LUAD epithelial cells by scRNA-seq analysis (Fig. 4B). Immunofluorescence staining further validated that FAM83A knockdown significantly decreased ErbB2 expression in PDO samples (Fig. 4C). Additionally, a positive correlation between FAM83A and ErbB2 intensities was observed in LUAD tissues (Fig. 4D). However, quantitative RT-PCR demonstrated that FAM83A knockdown did not alter ErbB2 mRNA expression in A549 and H1299 cells (Fig. S1A). Consistently, the TCGA database also showed no correlation between FAM83A and ErbB2 transcripts in LUAD tissues (Fig. S1B). These findings suggest that FAM83A regulates ErbB2 expression at the protein level, primarily involving protein synthesis (ribosome-mediated translation) and degradation. To explore the potential involvement of protein degradation in the regulation of ErbB2 expression by FAM83A, we used proteasome inhibitor MG132 and lysosomal inhibitor Chloroquine (CQ). MG132 prevented the downregulation of ErbB2 protein caused by FAM83A knockdown, while CQ had no effects (Fig. 4E, F). This indicates that FAM83A knockdown might promote ErbB2 degradation through the ubiquitin-proteasome system in LUAD. To further support the regulation of ErbB2 expression by FAM83A at the protein degradation level but not at the synthesis level, treatment of both A549 and H1299 cells with the protein synthesis inhibitor cycloheximide (CHX) for the indicated time confirmed that ErbB2 stability was significantly decreased in FAM83A knockdown cells (Fig. 4G). To investigate the potential effects of ErbB2 on CSC-like phenotypes mediated by FAM83A knockdown, ErbB2 was ectopically expressed in LUAD cells of control or FAM83A knockdown. Ectopic expression of ErbB2 rescued the inhibition of CD133 and CD44 expression mediated by FAM83A knockdown (Fig. 4H). Although FAM83A knockdown decreased CSC-like phenotypes, overexpression of ErbB2 significantly increased the spheroid formation capacity in cells with FAM83A knockdown, indicating that FAM83A knockdown decreased CSC-like phenotypes at least partly via suppression of ErbB2 (Fig. 4I, J).

### FAM83A interacted with ErbB2 and regulated its STUB1-mediated ubiquitin-proteasome degradation

**Fig. 5:**
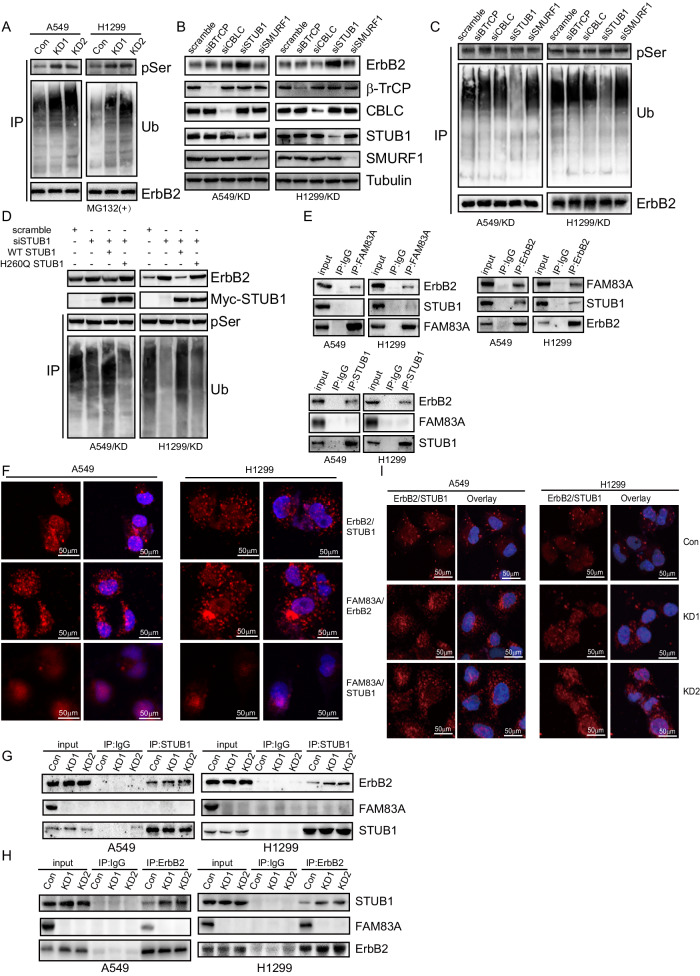
FAM83A interacted with ErbB2 and regulated its STUB1-mediated ubiquitin-proteasome degradation. **A** Co-immunoprecipitation (Co-IP) of serine phosphorylation and ubiquitination of ErbB2 in control or FAM83A knockdown LUAD cells incubated with MG132 for an additional 4 h, using ErbB2 as bait. **B** Western blot analysis of FAM83A knockdown LUAD cells transfected with scramble siRNA or siRNA specific against BTrCP, CBLC, STUB1, SMURF1, using the indicated antibodies. **C** Co-IP of serine phosphorylation and ubiquitination of ErbB2 in FAM83A knockdown LUAD cells transfected with scramble siRNA or siRNA specific against BTrCP, CBLC, STUB1, and SMURF1. **D** Western blot analysis of control or STUB1 knockdown LUAD cells with FAM83A knockdown, transfected with lentivirus containing empty, STUB1 (WT), STUB1 (H260Q) construct, using the indicated antibodies. **E** Co-IP of the indicated proteins in LUAD cells, using FAM83A, ErbB2, or STUB1 as bait. **F** DuoLink PLA analysis of the direct interaction of endogenous ErbB2 with STUB1, FAM83A with ErbB2 and FAM83A with STUB1 in LUAD cells. Representative images were provided. **G**, **H** Co-IP of the indicated proteins in LUAD cells with control or FAM83A knockdown, using STUB1 or ErbB2 as bait. **I** DuoLink PLA analysis of the direct interaction of endogenous ErbB2 with STUB1 in LUAD cells with control or FAM83A knockdown. Representative images were provided. Data represent means ± SD of three independent experiments; *P < 0.05; ns not significant.

Consistent with phosphorylation proteomics results, FAM83A knockdown increased the phosphorylation of ErbB2 and its ubiquitination in LUAD cells treated with MG132 (Fig. 5A). Several E3 ligases, including β-TrCP, CBLC, STUB1, and SMURF1, known for their role in ErbB2 ubiquitination, have been reported [26–29]. Silencing of the individual E3 ligase demonstrated that only STUB1 knockdown significantly increased ErbB2 expression in FAM83A knockdown LUAD cells (Fig. 5B). In addition, STUB1 knockdown significantly decreased ubiquitination, but not phosphorylation of ErbB2 in FAM83A knockdown cells (Fig. 5C). Furthermore, STUB1 knockdown increased ErbB2 expression and decreased its ubiquitination simultaneously. This effect was rescued by wild-type STUB1, but not the enzymatically inactive mutant H260Q (Fig. 5D). Co-immunoprecipitation (Co-IP) (Fig. 5E) and DuoLink proximity ligation assay (PLA) (Fig. 5F) demonstrated the interaction between ErbB2 and both FAM83A and STUB1, but not between FAM83A and STUB1. In addition, the interaction between STUB1 and ErbB2 was significantly upregulated in LUAD cells with FAM83A knockdown (Fig. 5G–I), indicating that FAM83A may suppress ubiquitination and proteasomal degradation of ErbB2 mediated by STUB1 via their interaction.

### FAM83A regulated CSC-like phenotypes of LUAD by interaction with ErbB2 via its Arg241 site

**Fig. 6:**
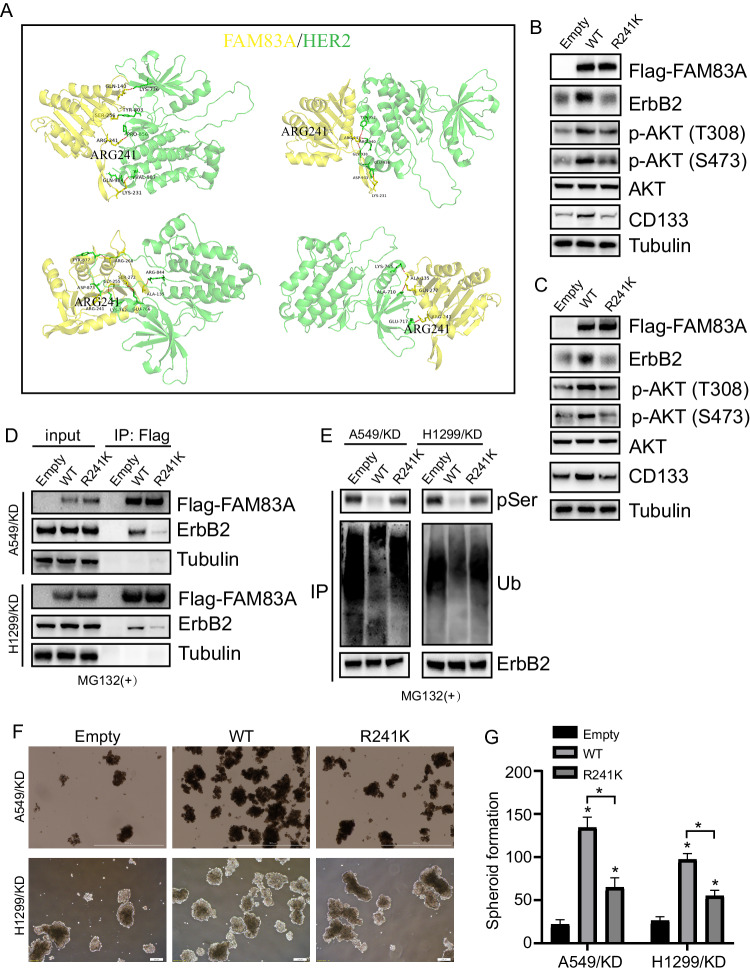
FAM83A regulated CSC-like phenotypes of LUAD by interaction with ErbB2 via its Arg241 site. **A** The interaction site of FAM83A with ErbB2 was analyzed by molecular docking. **B**, **C** FAM83A knockdown A549 (**B**) and H1299 (**C**) cells were infected with lentivirus containing empty, FAM83A (WT) or FAM83A (R241K) construct. Western blot analysis was performed using the indicated antibodies. **D** The interaction of FAM83A with ErbB2 was analyzed by Co-IP in the above cells incubated with MG132 for an additional 4 h, using Flag as bait. **E** Serine phosphorylation and ubiquitination of ErbB2 was performed by Co-IP in above LUAD cells incubated with MG132 for additional 4 h, using ErbB2 as bait. **F**, **G** Representative photographs of spheroids derived from the above LUAD cells and their numbers. Data represent means ± SD of three independent experiments; *P < 0.05; ns not significant.

The interaction sites between FAM83A and ErbB2 were predicted through molecular docking, and the Arg241 site of FAM83A attracted our interest due to its presence in multiple docking models (Fig. 6A). Wildtype (WT) or R241K mutant FAM83A was then introduced into FAM83A knockdown LUAD cells (Fig. 6B, C). WT FAM83A significantly increased levels of ErbB2, CD133, and phosphorylated AKT (Fig. 6B, C). In contrast, R241K mutant FAM83A did not alter ErbB2 and CD133 expression (Fig. 6B, C). While the effect was weaker compared to WT FAM83A, the R241K mutant FAM83A still increased the phosphorylation of AKT (Fig. 6B, C). It should be noted that the R241K mutant FAM83A almost completely blocked the interaction between FAM83A and ErbB2 (Fig. 6D). In addition, WT but not R241K mutant FAM83A significantly decreased phosphorylation and ubiquitination levels of ErbB2 in LUAD cells (Fig. 6E). Importantly, the recovery of WT FAM83A expression significantly promoted the spheroid formation of LUAD cells (Fig. 6F, G). Although the R241K mutant FAM83A also increased the spheroid formation capacity of LUAD cells with FAM83A knockdown, its effects were notably weaker than those of WT FAM83A (6F, G).

### Phosphorylation of ErbB2 promoted its interaction with STUB1

**Fig. 7:**
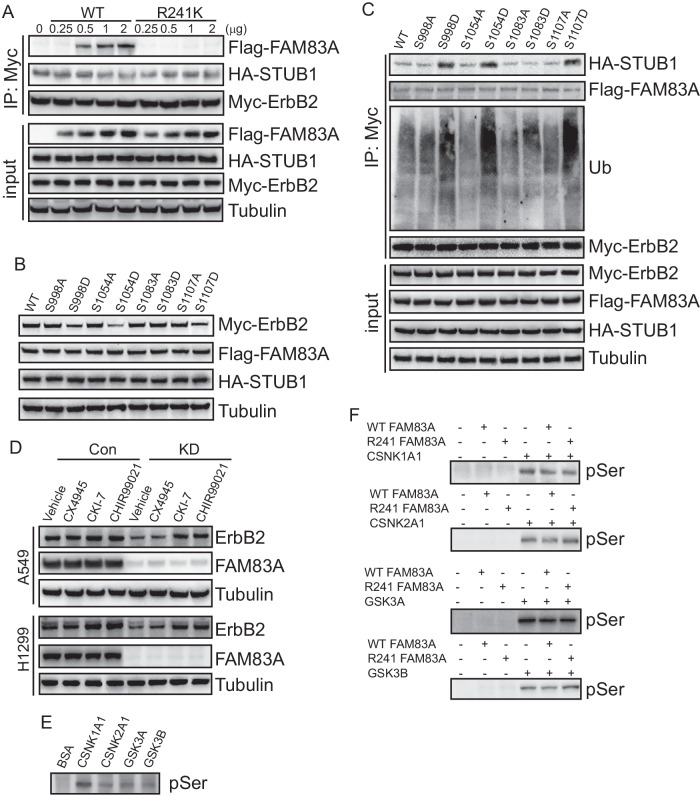
Phosphorylation of ErbB2 promoted its interaction with STUB1. **A** Co-IP of ErbB2 in HEK293 cells with 0, 0.25, 0.5, 1, 2 μg overexpressing exogenous WT or R241K mutant FAM83A respectively, using Myc as bait. **B** Western blot analysis of HEK293 cells infected with WT or phosphorylation mutant ErbB2 plasmid. The analysis was performed using the indicated antibodies. **C** Co-IP of ErbB2 in HEK293 cells overexpressing WT or phosphorylation mutant ErbB2. **D** Western blot analysis of control or FAM83A knockdown cells treated with CX4945, CKI-7, and CHIR99021. The analysis focused on the indicated protein expression levels. **E** In vitro kinase assays employing recombinant CSNK1A1, CSNK2A1, GSK3A, and GSK3B to measure the phosphorylation of ErbB2.The assessment was performed by Western blotting against pan-specific Ser phosphorylation antibodies. **F** Kinase assays employing recombinant CSNK1A1, CSNK2A1, GSK3A, and GSK3B with the phosphorylation of ErbB2 in FAM83A knockdown A549 cells overexpressing WT or R241K mutant FAM83A. The assessment was performed by Western blotting against pan-specific Ser phosphorylation antibodies. Data represent means ± SD of three independent experiments; *P < 0.05; ns not significant.

Considering the effect of the Arg241 site at FAM83A on ErbB2 expression, we speculated that FAM83A might compete with STUB1 to interact with ErbB2. Unexpectedly, there was no alteration in the interaction between ErbB2 and STUB1 in HEK293 cells with increasing expression of either WT or mutant FAM83A (Fig. 7A), ruling out competitive binding of ErbB2 by FAM83A and STUB1. Phosphorylation modification is well known to be closely related to ubiquitination [30]. Vectors containing WT, nonphosphorylation mutant (S998A, S1054A, S1083A, and S1107A), and phosphorylation mimic mutant (S998D, S1054D, S1083D, and S1107D) ErbB2 were constructed. When WT or mutant ErbB2 was cotransfected with FAM83A and STUB1, the phosphorylation mimic mutant S998D, S1054D, and S1107D of ErbB2 significantly decreased its protein expression. While the phosphorylation mimic mutant S1083D had no effect on the protein expression of ErbB2 (Fig. 7B). Furthermore, the interaction between ErbB2 and STUB1, as well as the ubiquitination of ErbB2, were upregulated by the phosphorylation mimic mutant S998D, S1054D, and S1107D of ErbB2 (Fig. 7C), indicating that phosphorylation of ErbB2 at the S998, S1054, and S1107 sites might be involved in the expression of ErbB2 regulated by STUB1. Bioinformatics predicted possible phosphorylation of ErbB2 by GSK3, CSNK1A1, and CSNK2A1 at these sites. Some kinase inhibitors, including CX4945 (inhibitor of CSNK2A1), CKI-7 (inhibitor of CSNK1A1), and CHIR99021 (inhibitor of GSK3A and GSK3B) were then used. CKI-7 or CHIR99021 significantly increased ErbB2 expression in FAM83A knockdown cells (Fig. 7D), indicating that CSNK1A1 and GSK3 might be involved in the phosphorylation and subsequent proteasomal degradation of ErbB2. In vitro phosphorylation confirmed that CSNK1A1, CSNK2A1, GSK3A, and GSK3B phosphorylated the recombinant fragment of ErbB2 containing amino acids 973-1146 (Fig. 7E). Surprisingly, neither WT nor mutant FAM83A altered the phosphorylation of the ErbB2 fragment by CSNK1A1, CSNK2A1, GSK3A, and GSK3B (Fig. 7F).

### Dephosphorylation of ErbB2 by CALN was implicated in its expression regulation by FAM83A

**Fig. 8:**
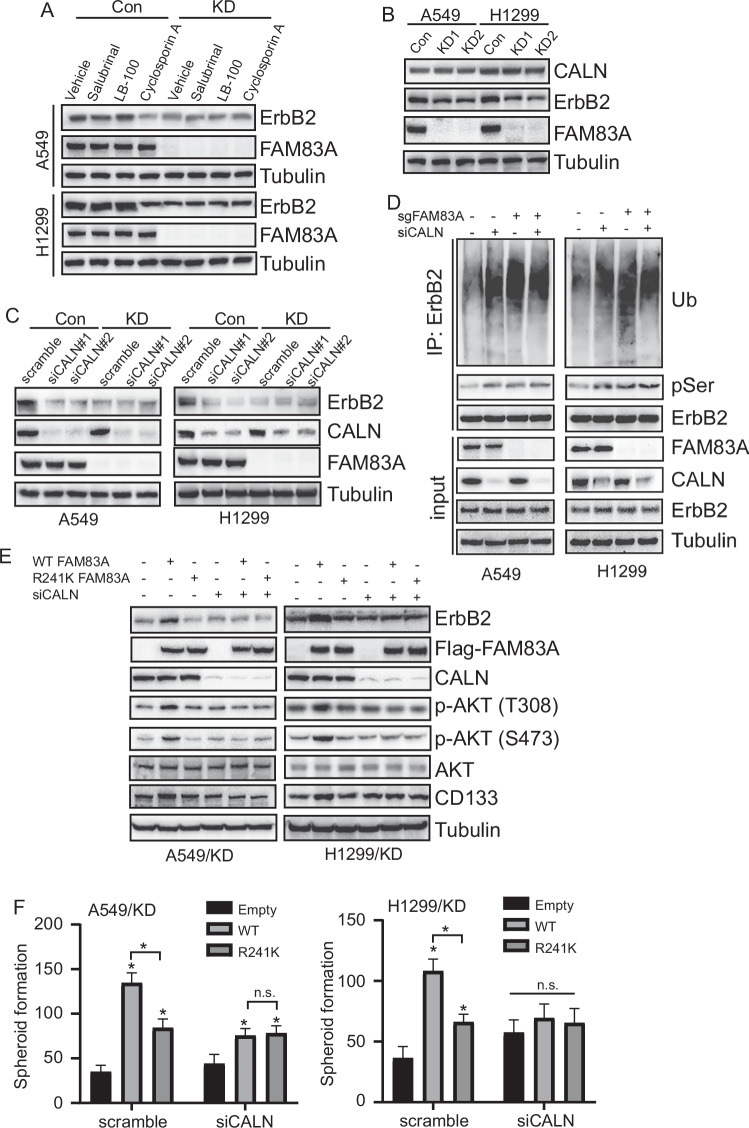
Dephosphorylation of ErbB2 by calcineurin was implicated in its expression regulation by FAM83A. **A** Western blot analysis of control or FAM83A knockdown cells treated with Salubrinal, LB100, and Cyclosporin A. The analysis focused on the indicated protein expression levels. **B** Western blot analysis of the indicated protein expression levels in control or FAM83A knockdown cells. **C** Western blot analysis of control or FAM83A knockdown LUAD cells transfected with scramble siRNA or siRNA specific against CALN. The analysis focused on the indicated protein expression levels. **D** Co-IP of serine phosphorylation and ubiquitination of ErbB2 in the above LUAD cells, using ErbB2 as bait. **E** Western blot analysis of FAM83A knockdown cells with overexpressing WT or R241K mutant FAM83A, transfected with scramble siRNA or siRNA specific against CALN. The analysis focused on the indicated protein expression levels. **F** Quantification of spheroids derived from the above LUAD cells. Data represent means ± SD of three independent experiments; *P < 0.05; ns not significant.

As FAM83A showed no effect on the phosphorylation of ErbB2 by kinases, we speculated that certain phosphatases might be involved. Phosphatase inhibitors, such as Salubrinal (inhibitor of PP1), LB100 (inhibitor of PP2A), and Cyclosporin A (inhibitor of PP2B, also known as CALN), were then employed. Interestingly, only Cyclosporin A, but not Salubrinal or LB100, significantly decreased ErbB2 expression in both control A549 and H1299 cells (Fig. 8A). Notably, the impact of Cyclosporin A on ErbB2 expression was not evident in FAM83A knockdown cells (Fig. 8A), suggesting the implication of FAM83A in the dephosphorylation of ErbB2 by CALN (Fig. 8A). FAM83A knockdown had no observable effect on CALN expression in LUAD cells (Fig. 8B). Further confirmation was obtained by CALN knockdown using siRNA specific against CALN (siCALN) in both control and FAM83A knockdown cells. CALN knockdown significantly decreased ErbB2 expression in control cells compared with FAM83A knockdown cells (Fig. 8C). In meanwhile, the phosphorylation and ubiquitination of ErbB2 were significantly upregulated by CALN knockdown in control cells, while no obvious effects were observed in FAM83A knockdown cells (Fig. 8D). Moreover, WT FAM83A, but not the R241K mutant, significantly increased ErbB2, CD133, and phosphorylated AKT in FAM83A knockdown cells, and this effect was blocked by CALN knockdown (Fig. 8E). CALN knockdown also significantly inhibited the promotion of spheroid formation by WT FAM83A in LUAD cells (Fig. 8F). It is noteworthy that, compared with the R241K mutant FAM83A, WT FAM83A exerted a stronger promotional effect on spheroid formation in control LUAD cells. However, when CALN was knocked down, similar effects were observed in cells transfected with both WT and R241K FAM83A (Fig. 8F).

## Discussion

Lung cancer poses a significant global health threat, ranking first in mortality among all types of tumors, with over 40% of cases being LUAD [31, 32]. With the development of molecular medicine and targeted therapy, especially the use of tyrosine kinase inhibitors (TKIs), the survival of patients with oncogene-driven advanced-stage LUAD has been prolonged [5]. Nevertheless, the utility of targeted therapy has reached a plateau, and the overall prognosis of LUAD remains relatively poor [2]. Thus, there is an urgent need to identify more critical molecular events underlying tumorigenicity, which could pave the way for more effective therapeutic strategies for LUAD.

Tumor progression has been intricately linked to the presence of CSCs within the tumor bulk, playing pivotal roles in various malignant phenotypes such as metastasis, recurrence, and drug resistance. A previous study reported significant overexpression of FAM83A in pancreatic cancers, indicating its involvement in CSC-like phenotypes [23]. Consistent with these findings, our study revealed upregulation of FAM83A in LUAD clinical samples, with a correlation to worse overall survival. FAM83A knockdown substantially suppressed several CSC-like phenotypes, including spheroid formation capacity, expression of CSC marker, and xenograft tumor formation in LUAD cell lines and PDO models. Utilizing a global proteomics method, our study further demonstrated a positive correlation between ErbB2 and FAM83A in LUAD cells and tissues.

The human epidermal growth factor receptor (HER) tyrosine kinase family comprises ErbB1/EGFR/HER1, ErbB2/HER2, ErbB3/HER3, and ErbB4/HER4 [33]. These receptors play important roles in cellular processes, including growth, proliferation, differentiation, and survival [34]. Specifically, ErbB2, lacking any known EGF family ligand, acts as an oncogenic driver by heterodimerizing with other HER family members to mediate cell proliferation through the Ras-Raf-MAPK and PI3K/AKT signaling pathways [35]. ErbB2 is membrane receptor tyrosine kinase involved in HER signaling to which various ligands can be attached, leading to PI3K/AKT activation [36]. Hyperactivation of pro-survival and pro-proliferative pathways related to ErbB2, such as PI3K/AKT leads to cancer initiation, which affects mitochondria [37]. Besides, studies have shown that ErbB2 recruits AKT1 to disrupt STING signaling and suppress antiviral defense and antitumor immunity [38]. Importantly, our data indicate that FAM83A knockdown decreases the expression of ErbB2 and the activity of its related AKT signaling pathway in LUAD cells. ErbB2 amplification and mutations are two distinct gene alterations that are observed in ~2–10% of NSCLC. ErbB2 mutation is recognized as an oncogenic driver in LUAD, but the role of ErbB2 amplification remains uncertain [39]. ErbB2 has been shown to participate in the pathophysiology of LUAD, implicating its role as an actionable driver in lung cancers and correlating with poor prognosis [34]. Our data indicate that the inhibition of CSC-like phenotypes by FAM83A knockdown is rescued by ErbB2 overexpression. The latest report indicated that patients with ErbB2-amplified LUAD exhibit better immunogenicity and an inflamed time among ErbB2-aberrant tumors [40]. However, the underlying mechanism of ErbB2 amplification in LUAD remains unclear.

The current study demonstrates that FAM83A regulates ErbB2 expression at the posttranslational degradation level. Two primary intracellular protein degradation pathways, namely the ubiquitin-proteasome and autophagy-lysosome systems, play crucial roles in this process. Our investigation revealed that FAM83A knockdown enhances STUB1-mediated ubiquitin-proteasome degradation of ErbB2. Previous studies have found that STUB1 regulates a variety of oncogenic proteins, including ErbB2 in breast cancer [41]. Serving as a chaperone-dependent E3 ubiquitin ligase, STUB1 comprises three distinct domains: an N-terminal tetrapeptide repeat (TRP) domain, a middle coiled-coil (CC) domain, and a C-terminal U-box domain [26]. Typically, STUB1 regulates the degradation of target substrate proteins via K48-linked ubiquitination. However, in a few cases, it may also be K63- or K27-linkage [41]. Nevertheless, further investigation is warranted to elucidate the specific mechanism by which STUB1 ubiquitinates ErbB2 and subsequently targets it for degradation via the proteasomal pathway.

The present study unveils the interaction of FAM83A with ErbB2 at the Arg241 site, which inhibits the recruitment of ErbB2 to STUB1 and contributes to CSC-like phenotypes in LUAD. Despite the interaction between FAM83A and ErbB2, our observations suggest that FAM83A does not compete with STUB1. Phosphorylation and ubiquitination often exhibit extensive crosstalk during the ubiquitin-proteasome pathway [42]. This regulation is always achieved through the phosphorylation of either the substrate or the ligase itself to promote substrate recognition by ubiquitin ligases [30, 38, 43]. As a classical posttranslational modification of proteins, protein kinases, and phosphatases play important roles in dynamically regulating protein phosphorylation [44]. The spheroid formation capacity of LUAD cells with the R241K mutant FAM83A was notably weaker than those of WT FAM83A, indicating the R241 site might play an important role in the regulation of CSC-like phenotypes by FAM83A. However, the R24lK mutation did not completely eliminate the effect of FAM83A on the spheroid formation capacity, suggesting that there may be alternative mechanisms involved. In this work, we observed that the phosphorylation of ErbB2, regulated by protein phosphatase type 2B (PP2B, also known as CALN), promotes its interaction with STUB1, which is essential for its expression regulated by FAM83A and the maintenance of CSC-like phenotype. Therefore, our data suggest that blocking the interaction between FAM83A and ErbB2 at the Arg241 site holds strong potential as a treatment target for LUAD.

**Fig. 9:**
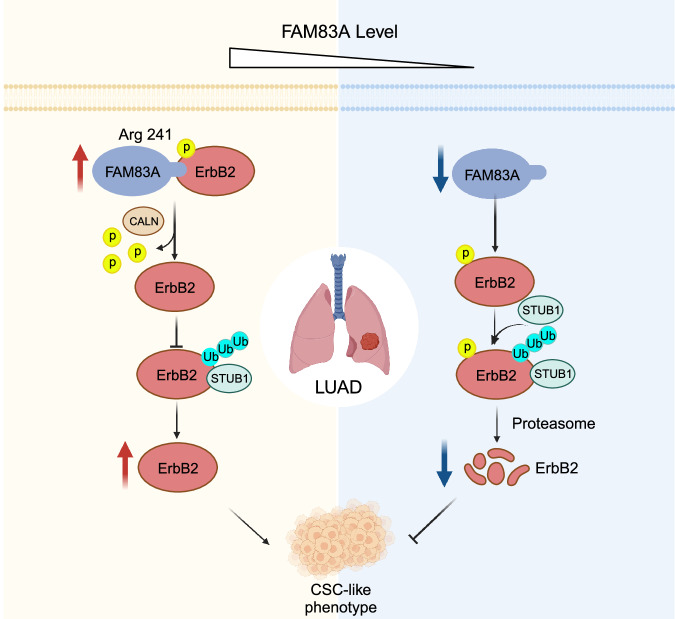
Working model.

In conclusion, this study elucidated the role of FAM83A as a tumor-promoting gene influencing the CSC-like phenotype of LUAD. Moreover, FAM83A was found to interact with ErbB2 at the Arg241 site, thereby inhibiting STUB1-mediated ubiquitin-proteasome degradation of ErbB2. This interaction was related to the dephosphorylation of ErbB2, regulated by CALN. Consequently, these molecular events activated the classical AKT pathway, contributing to the progression of LUAD (Fig. 9).

However, this study has its limitations. For example, the mechanism by which FAM83A recruits ErbB2 at the Arg241 site remains unresolved. Future research should delve into the potential mechanisms underlying how the interaction between FAM83A and ErbB2 regulates the dephosphorylation of ErbB2 mediated by CALN, aiming to unveil a more comprehensive regulatory network. Despite these limitations, the identified mechanism of FAM83A, acting as a tumor-promoting gene, influencing the CSC-like phenotype of LUAD with respect to the expression of ErbB2, contributes to a better understanding of the specific role of FAM83A in LUAD. This insight may aid in the screening of potential molecular targets for prognosis and treatment.

## Materials and methods

### Cell culture

A549 and H1299 human LUAD cell lines were acquired from the American Type Culture Collection (ATCC, USA) and cultured in RPMI1640 medium (Biological Industries, Israel), supplemented with 10% fetal bovine serum (Biological Industries) and 1% penicillin-streptomycin (Sigma-Aldrich, USA). The Cells were maintained in a humidified incubator at 37 °C with 5% CO_2_ and utilized during the logarithmic phase of growth.

### Knockdown of FAM83A by CRISPR/Cas9

A dual gRNA approach was used to knock down FAM83A by the CRISPR/Cas9 system. To facilitate the selection of positive clones, a donor vector was generated, ensuring that the targeting sequence was replaced by a marker gene (PU, the puromycin-resistance gene) upon integration into the genomic DNA through homologous recombination. The dual gRNA construct, carrying Cas9 and the donor vector, was introduced into lung cancer cells by infection. The target sequences were AGAAGTTCATCATCTCGGAC and GAGATATACTGTGCCAAGTC. An empty dual gRNA vector served as a control. After 1 week, the infected cells were subjected to puromycin selection. Initial identification of knockdown was carried out by western blot.

### RNA interference

Specific siRNA oligos against human SMURF1, STUB1, CBLC, and β-TrCP were synthesized by GenePharm (Shanghai, China). Their sequences were as follows: SMURF1 siRNA: 5′-GCAUCGAAGUGUCCAGAGAAG-3′, STUB1 siRNA: 5′-GGCAAUCGUCUGUUCGUGGGCCGAA-3′, CBLC siRNA: 5’-CGUGUCCAUCUUCGAGUUCGA-3’, β-TrCP siRNA: 5’-TTGGATCCGCCACCATGGACCC GGCCGAGGCGGTGCTGC-3’, control siRNA: 5′-UUCUCCGAACGUGUCACGU-3′. Additionally, the negative control RNA duplexes and human CALN siRNAs were purchased from Hechuang Biotechnology Co., Ltd. (Guangzhou, China). Their sequences were as follows: control siRNA: 5′-UUCUCCGAACGUGUCACGU-3′, CALN siRNA1: CCAAGUUGUCGACGACCGA, CALN siRNA2: AGUGUUGCAUUGAGAAUAA. Cells were transfected with siRNAs using Lipofectamine® RANiMAX reagent (13778150, Thermo Scientific) following the manufacturer’s instructions.

### Nude mouse transplantation tumor experiment

BALB/c-nu/nu female mice (4 ± 1 weeks old, 19 ± 5 g), purchased from Liaoning Changsheng Biotechnology Co., Ltd (China), were housed in plastic polyvinyl chloride cages (*n* = 3 per cage) equipped with sealed air filters, animal isolators, and a laminar airflow device. The cages were kept in a laminar airflow chamber at 24 ± 1 ◦C with alternating 12-h periods of light and darkness. All in vivo experiments were conducted in accordance with the approved protocol from the Experimental Animal Center of China Medical University and adhered to relevant policies. The mice were randomly divided into eight groups. For the subcutaneous xenograft model, 5 × 10^3^, 5 × 10^4^, 5 × 10^5^, or 5 × 10^6^ transfected A549 cells were suspended in 100 μl of sterile phosphate-buffered saline (PBS) and then subcutaneously injected into the bilateral backs of nude mice (*n* = 5 per group). Tumor formation was observed dynamically. Twenty-eight days later, the mice were sacrificed by cervical dislocation, and the subcutaneous tumors were examined. All animal experiments complied with the guidelines of the Institutional Animal Care Committee of China Medical University.

### Western blot

After collecting the cells, total protein was extracted with lysis buffer containing 20 mM Tris-HCl, 150 mM NaCl, 2 mM EDTA, 1% Triton X-100, and a protease inhibitor cocktail (Sigma-Aldrich). Subsequently, protein concentrations were measured using the BCA Protein Assay reagent (Thermo Fisher Scientific, USA) before being adjusted conformably among samples. Next, 20 μg of total protein was separated by 12% SDS–PAGE at 200 V for 60 min. Resolved proteins were transferred to polyvinylidene difluoride membranes (EMD Millipore, Germany). After blocking with 3% skimmed milk, the membranes were incubated in sequence with primary antibodies and horseradish peroxidase (HRP) conjugated secondary antibodies. Immunoreactive bands were visualized by hypersensitive chemiluminescence reagents (Tanon, China). The primary antibodies targeted FAM83A (SAB2108978, 1:1000), ErbB2 (SAB5700151, 1:1000), phosphorylated ErbB2 (p-ErbB2, Y1221/1222, SAB5700388, 1:1000), Tubulin (T3526, 1:5000) were all from Sigma-Aldrich. The primary antibodies targeted S6K1 (9202 S, 1:1000), phosphorylated S6K1 (p-S6K1, T389, 9234 S, 1:1000), phosphorylated AKT (p-AKT, T308, 13038 S, 1:1000), AKT (9272 S, 1:1000), phosphorylated AKT (p-AKT, S473, 4060 S, 1:2000), β-TrCP (4394 S, 1:1000), CBLC (2747 S, 1:1000), STUB1 (2080 S, 1:1000), SMURF1 (2174 S, 1:1000) and CD44 (37259, 1:1000) were all from Cell Signaling Technology. The primary antibodies targeted Ubiquitin (Ub, YT5498, 1:1000) were from Immunoway. The primary antibodies targeted c-Myc (MA1-980, 1:1000), DYKDDDDK Tag (Flag, MA1-91878, 1:1000) and HA-Tag (26183, 1:1000) were all from Invitrogen. The primary antibodies targeted CD133 (sc-365537, 1:1000) and CALN (sc-17808, 1:1000) were from Santa Cruz Biotechnology. The primary antibody targeted pan-specific Ser phosphorylation (pSer, ab117253, 1:1000) was obtained from Abcam.

### Quantitative PCR assay

TRIzol Reagent (Invitrogen, Carlsbad, CA, USA) was used to extract total RNA from cells. Then the RNA was reverse-transcribed with Moloney murine leukemia virus reverse transcriptase (Promega, Madison, WI, USA). Quantitative PCR was performed using the SYBR Ex Taq Kit (Takara, Japan). The expression levels of ErBb2 were normalized to the glyceraldehyde-3-phosphate dehydrogenase expression. The following gene-specific primers were used: ErbB2: 5′-GATGCTGTGGACCAAGTGAA-3′, 3′-TGCTCATCAATGCACCAAAT-5′; GAPDH: 5′-GAGTCAACGGATTTGGTCGT-3′, 3′-TGGAAGATGGTGATGGGATT-5′. Results were normalized by those of GAPDH and presented as ratio vs. vehicle-treated control. Three independent quantitative RT-PCR experiments were done in triplicate.

### Spheroid formation assay

Cells were digested with 0.25% trypsin, followed by centrifugation at 800 rpm for 5 min, and resuspended in serum-free DMEM/F12 medium (Biological Industries, Israel). Cells (3 × 10^4^) were seeded into ultralow-attachment six-well plates (Corning, USA) and cultured in 3 ml of serum-free DMEM/ F12 medium supplemented with 20 mg/ml human recombinant epidermal growth factor, 5 μg/ml insulin (both from Sigma-Aldrich), and 2% B27 (Invitrogen). During the culture period, the medium was replaced every 3 days. After 10–14 days, spheroids were photographed using a Cytation 5 Cell Imaging Multi-Mode Reader (BioTek Instruments, USA). Spheroids exceeding 50 μm in diameter were counted.

### Immunofluorescence (IF) staining

Paraffin sections, after dewaxing and hydration, were subjected to antigen retrieval by a high-pressure method and permeabilized with 0.1% Triton X-100 in PBS. The sections were blocked with PBS containing 1% bovine serum albumin for 1 h at room temperature. Immunostaining was then performed using primary antibodies against FAM83A (Invitrogen, PA5-46441, 1:100) and ErbB2 (Invitrogen, MA5-13675, 1:100), followed by fluorescently tagged secondary antibodies (Abcam, ab150113 and ab150078, 1:500). Finally, coverslips were mounted with DAPI and photographed using a confocal microscope.

### Tissue microarray and immunohistochemical staining

LUAD tissue microarray sections were purchased from Shanghai Outdo Biotech (China). Immunohistochemical staining was performed on the sections using antibodies against FAM83A (Invitrogen, PA5-46441). The sequential steps performed on the tissue sections included antigen retrieval, inhibition of endogenous peroxidase activity with 3% hydrogen peroxide, blocking of non-specific binding sites with 3% normal goat serum, incubation with primary antibodies, washing steps, incubation with horseradish peroxidase-conjugated IgG secondary antibody, and reaction in freshly prepared 3,3′-diaminobenzidine. A semiquantitative H-score was used to assess FAM83A expression levels. FAM83A expression levels were semiquantitatively evaluated using an H-score, calculated as the product of staining intensity (0, negative; 1, weak staining; 2, moderate staining; 3, strong staining) and the corresponding distribution areas (0%-100%). The primary antibodies targeted CK7 (ZM-0071) were from Beijing Zhongshan Jinqiao Biotechnology Co., Ltd.

### Generation and expansion of patient-derived xenografts (PDXs) and patient-derived organoids (PDOs)

The established PDXs originated from a patient diagnosed with primary LUAD, and all patients involved in this study provided written informed consent. The tumor tissue underwent PBS washing and was then cut into suitable parts. For xenograft implantation, needle biopsies were subcutaneously implanted in BALB/c-nu/nu female mice (4 ± 1 weeks old, 19 ± 5 g) under anesthesia (Domitor® 0.5 mg/kg, Dormicum 5 mg/kg, Fentanyl 0.05 mg/kg).

To propagate PDXs models, resected tumors were digested in 25 ml collagenase from clostridium (1 mg/ml) in PBS for 45 min at 37 °C, with occasional agitation by vortexing to dissociate tissue. Tissue fragments were then diluted 1:1 with RPMI media containing 100 U/ml penicillin and 100 μg/ml streptomycin, strained through a 70-μm strainer, and centrifuged at 1250×*g* for 10 min. The resulting pellet was resuspended in 20 ml of RPMI and strained through a 40-μm strainer. Dissociated cells were suspended in Matrigel and HITES media at a concentration of 1 × 10^6^ cells per 100 μl final volume per injection site. The generation and characterization of the PDO models have been described previously [45].

### DuoLink proximity ligation assay

The duoLink proximity ligation assay enables the observation of protein–protein interactions within the cell, providing clear visual signals. The signal is generated only if the proteins of interest are located within 40 nm, thereby detecting interaction. All procedures were performed according to the manufacturer’s instructions (Millipore Sigma, USA). To estimate the PLA signals, the image data were analyzed for the mean fluorescence intensity of the PLA signals and/or the total number of PLA signals per cell or per area within the cell. Quantification was then reported relative to technical and/or biological controls within a given experiment.

### Co-immunoprecipitation (Co-IP)

Cell lysates were pre-cleared with Protein-A/G beads (biomake, B23201) and kept on a rotator for 1 h at 4 °C. Following centrifugation, 2 μg of primary antibody or the corresponding IgG was added to the pre-cleared lysates and incubated on a rotator overnight at 4 °C. Subsequently, 25 μl of Protein-A/G beads were added and kept on a rotator for 2 h at 4 °C. The immunoprecipitates were washed three times with lysis buffer and then analyzed by Western blot analysis.

### In vitro kinase assay

For the in vitro kinase assay, kinase reactions were performed in 30 μL kinase buffer (20 mM Tris-HCl, pH 7.5,100 mM NaCl, 10 mM MgCl_2_, 1 mM DTT, 0.2 mM ATP, 5 mM EDTA). The purified kinase and substrate proteins (1: 10, 10 μg in total) in the kinase buffer were incubated with gentle shaking for 4 h at room temperature. Following the incubation, the proteins were separated with 10% SDS–PAGE, and the phosphorylation of proteins was detected by immunoblotting with pan-specific Ser phosphorylation antibodies.

### Molecular docking

The chemical structure of proteins was retrieved from the UniProt Database. The proteins were pretreated with MOE by adding hydrogen, assigning partial charges and protonation states, removing water molecules, and energy minimization with the OPLS-2005 force field. The original location of the protein-ligand was used as the ligand space, and the original ligand was removed to generate protein docking files. Potential ionization states were obtained using Epik at pH 7.0. Subsequently, all proteins were docked into the binding site in terms of the standard precision (SP) mode of Glide. The docking results were visualized using PyMoL 2.5 and LigPlus 2019.

### Statistical analyses

The experimental data are presented as mean ± standard deviation (X ± SD) and were statistically analyzed using SPSS 23.0 (IBM, USA). According to the normality and variance homogeneity of the data, one-way analysis of variance (one-way ANOVA) and Dunnett’s post hoc test were adopted to analyze group comparisons. Additionally, the log-rank test was used to assess overall survival distribution, the chi-square (*χ*^2^) test was applied for comparing composition ratios, and Pearson’s correlation test was used to calculate the coefficient of association. In vitro experiments were repeated three times independently. A *P* value <0.05 was considered statistically significant. The sample size was chosen by PASS 14.0 (NCSS, USA).

## Supplementary information

Related Manuscript File-Raw data for WB

Supplementary material-Figure S1

Figure S1 figure legends

## Data Availability

The data supporting the findings of this study are available from the corresponding author upon reasonable request.
