# Correction: Promotion of stem cell-like phenotype of lung adenocarcinoma by FAM83A via stabilization of ErbB2

**DOI:** 10.1038/s41419-026-08535-1

**Published:** 2026-04-02

**Authors:** Ye Yuan, Liang Hao, Jing-Shan Huang, Fu-Ying Zhao, Ying-Hua Ju, Jia-Mei Wang, Ting Zhang, Bai-Qiang Li, Zhan-Wu Yu

**Affiliations:** 1 Central Laboratory, Cancer Hospital of China Medical University, Liaoning Cancer Hospital & Institute, Cancer Hospital of Dalian University of Technology, Shenyang, 110042 China; 2 Department of Biochemistry & Molecular Biology, China Medical University, Shenyang, 110026 China; 3 Department of Chemistry, School of Forensic Medicine, China Medical University, Shenyang, 110026 China; 4 Department of Thoracic Surgery, the Shengjing Hospital, China Medical University, Shenyang, 110001 China; 5 Department of Laboratory Medicine, the 1st affiliated hospital, China Medical University, Shenyang, 110001 China; 6 Department of Thoracic Surgery, Cancer Hospital of China Medical University, Liaoning Cancer Hospital & Institute, Cancer Hospital of Dalian University of Technology, Shenyang, 110042 China

**Keywords:** Prognostic markers, Cancer stem cells

Correction to: *Cell Death & Disease* 10.1038/s41419-024-06853-w, published online 28 June 2024

During a post-publication data review, we identified an error in the manuscript. Specifically, in Figure 8D (the image from the second line in the rightmost column), an incorrect image was inadvertently included. This occurred due to an oversight during the final assembly of the figure, combined with a mistake in our image file management.

This error relates solely to the presentation of the image and does not affect the quantitative data, overall experimental results, or conclusions of the study. To rectify this, we have prepared a corrected version of Figure 8D, generated directly from the original source data, which accurately reflects our experimental findings, and have also included representative images from the three replicate experiments.

Incorrect figure 8
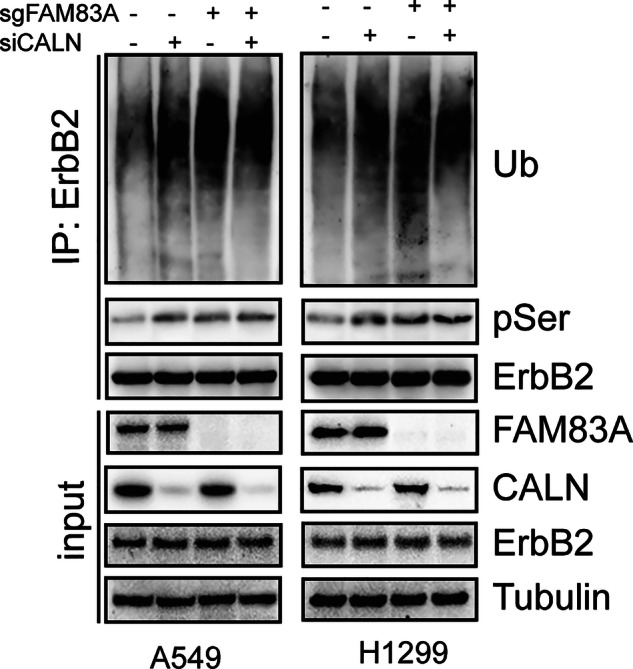


Correct figure 8
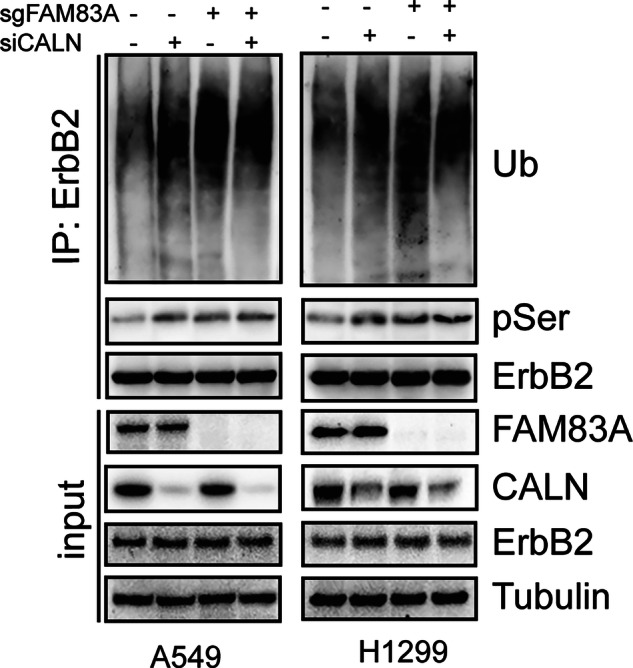


The original article has been corrected.

