# Supplementary material for: Promotion of stem cell-like phenotype of lung adenocarcinoma by FAM83A via stabilization of ErbB2
Source: Cell Death Dis. 2024 Jun 28;15(6):460. doi: 10.1038/s41419-024-06853-w (PMC11213963; doi:10.1038/s41419-024-06853-w)

FAM83A expression of 41 patients' tissue in Figure 1E

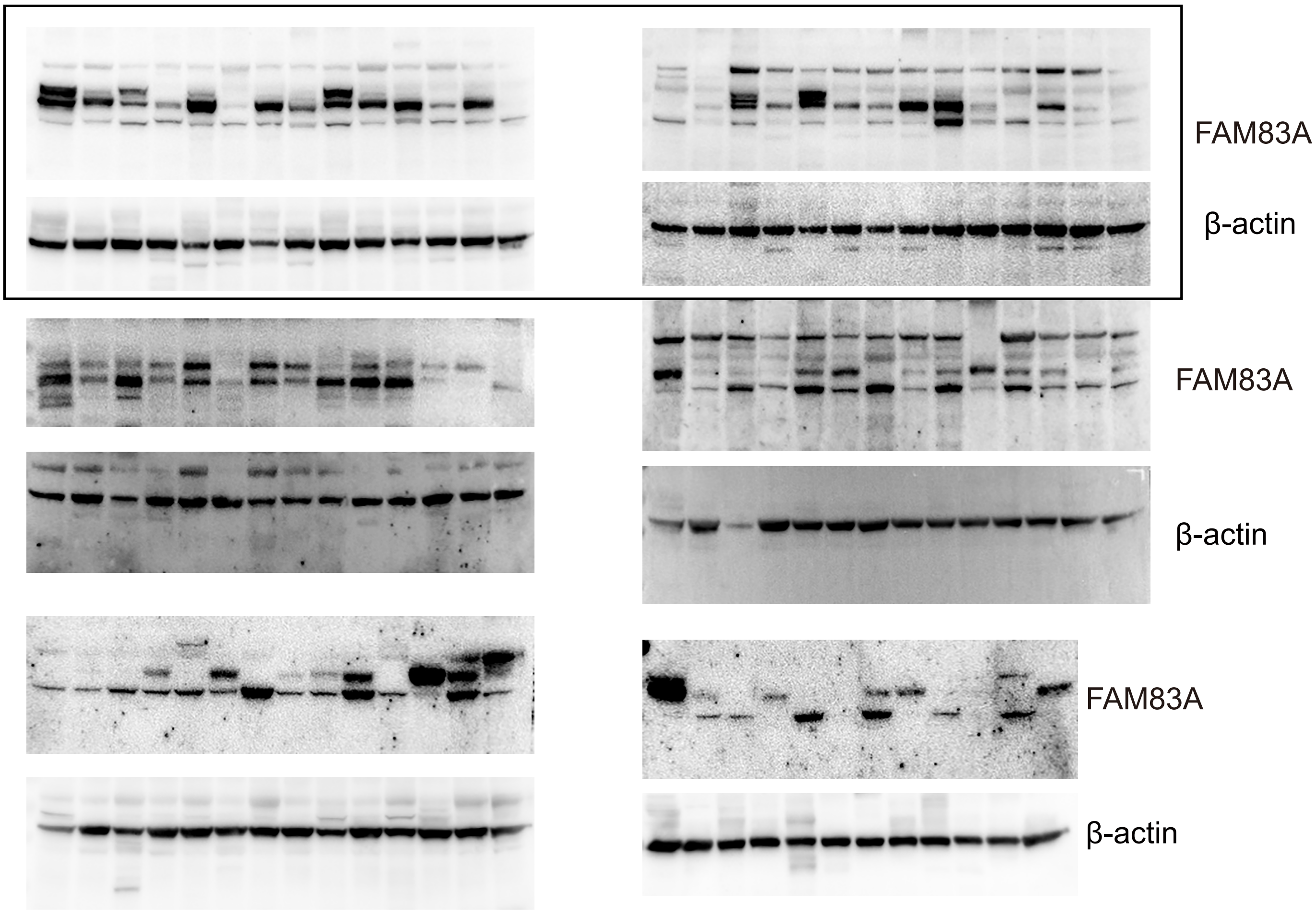

Figure 1

E

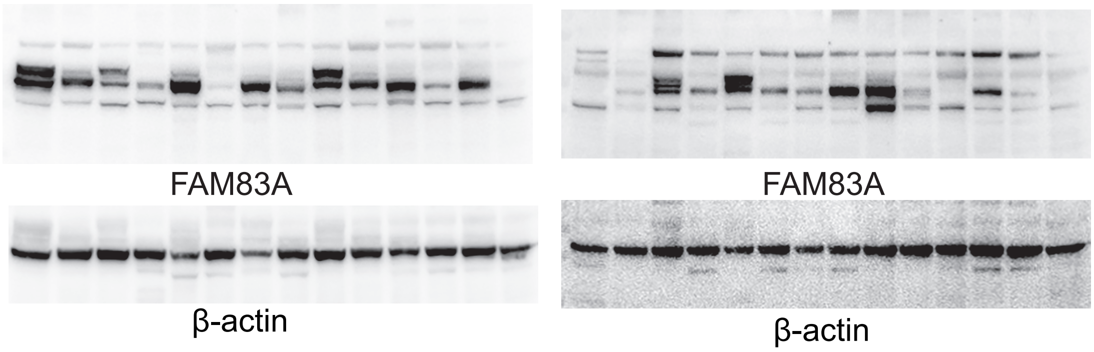

Figure 2

A

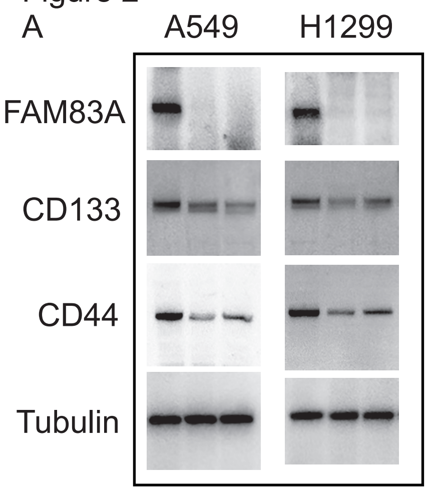

F

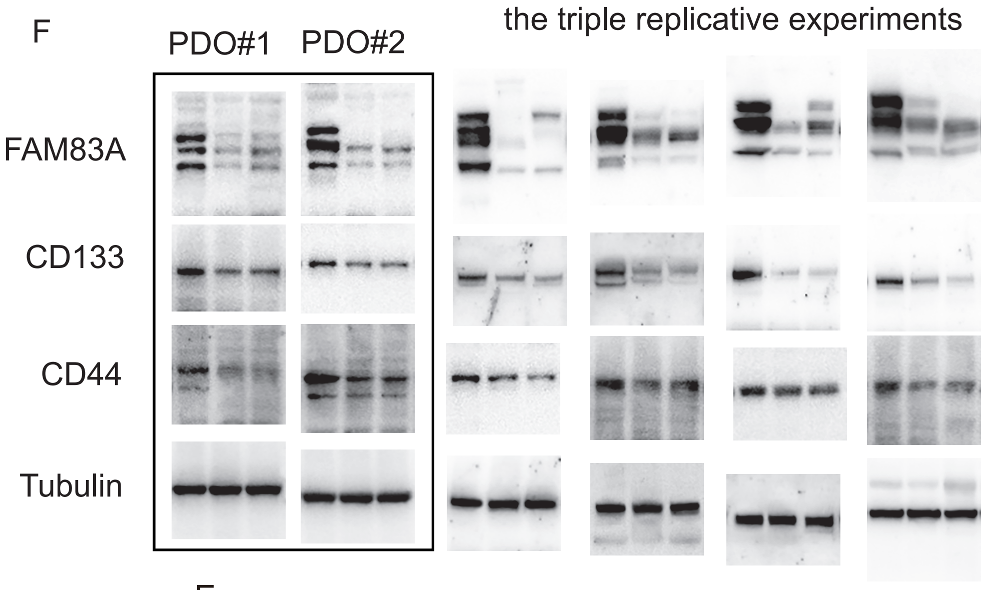

Figure 4

A

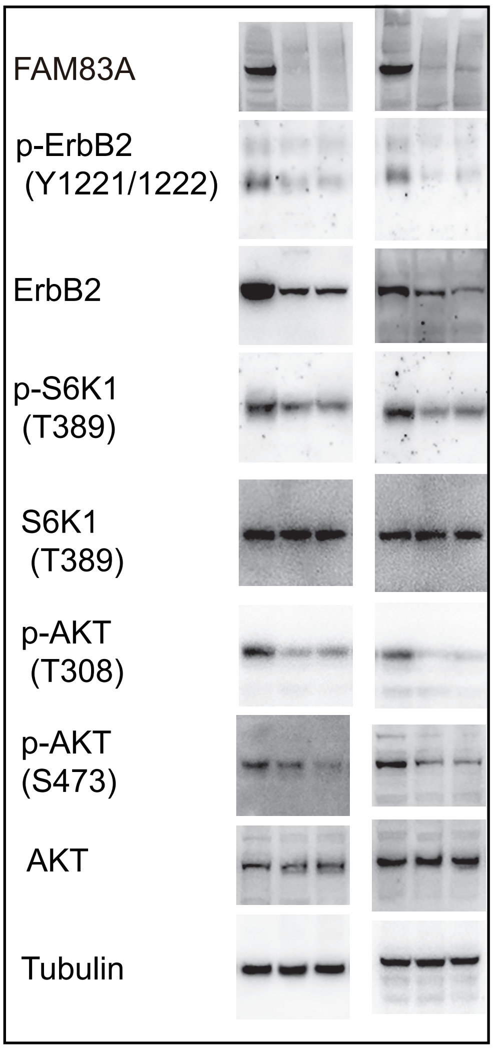

E

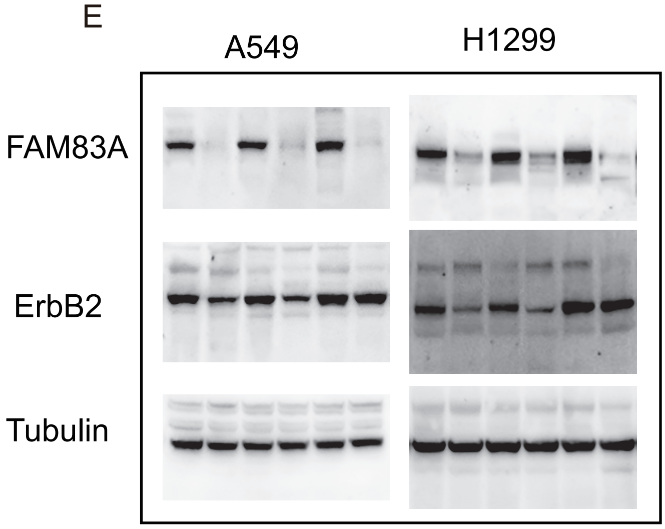

Figure 4

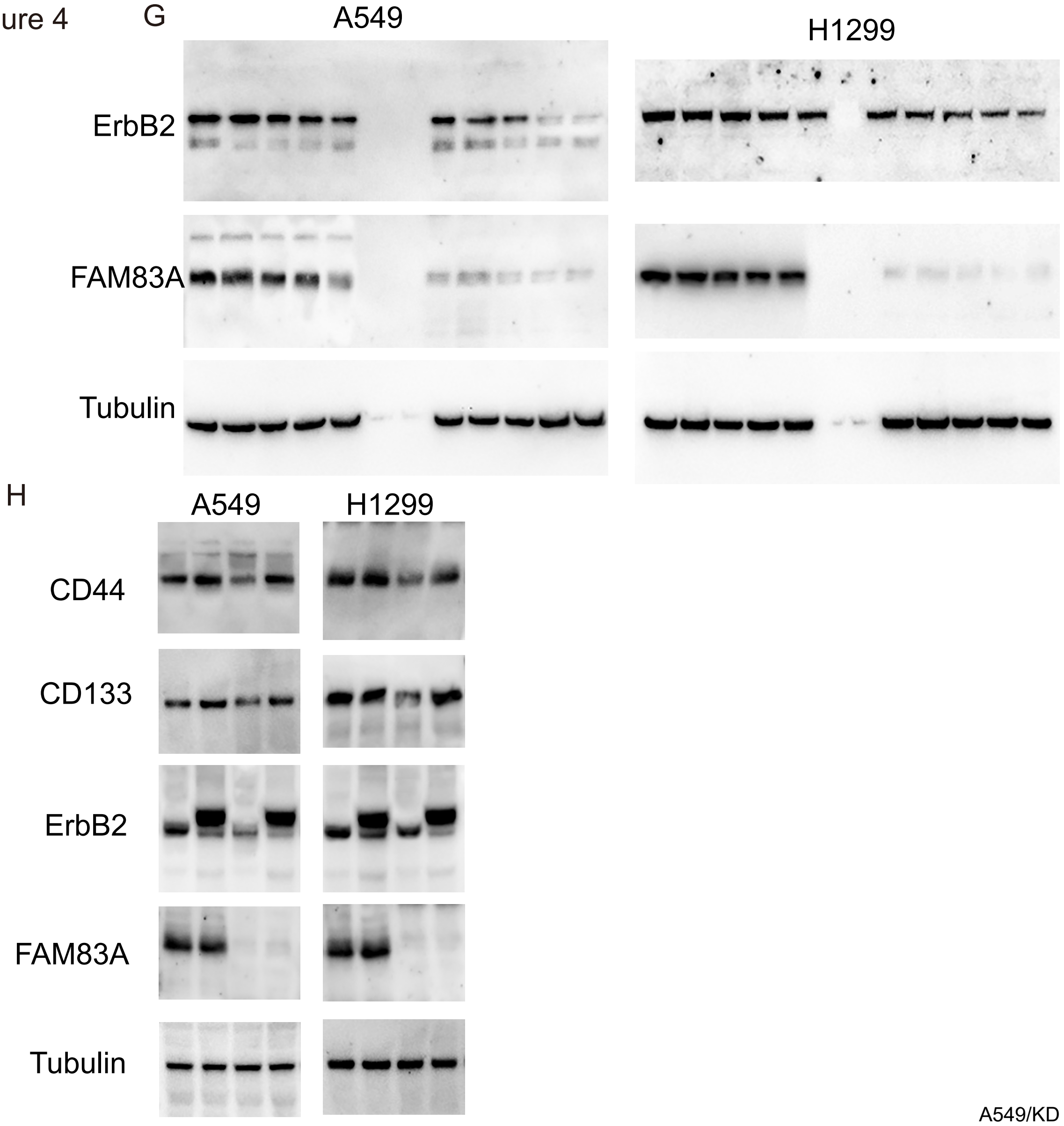

Figure 5

A

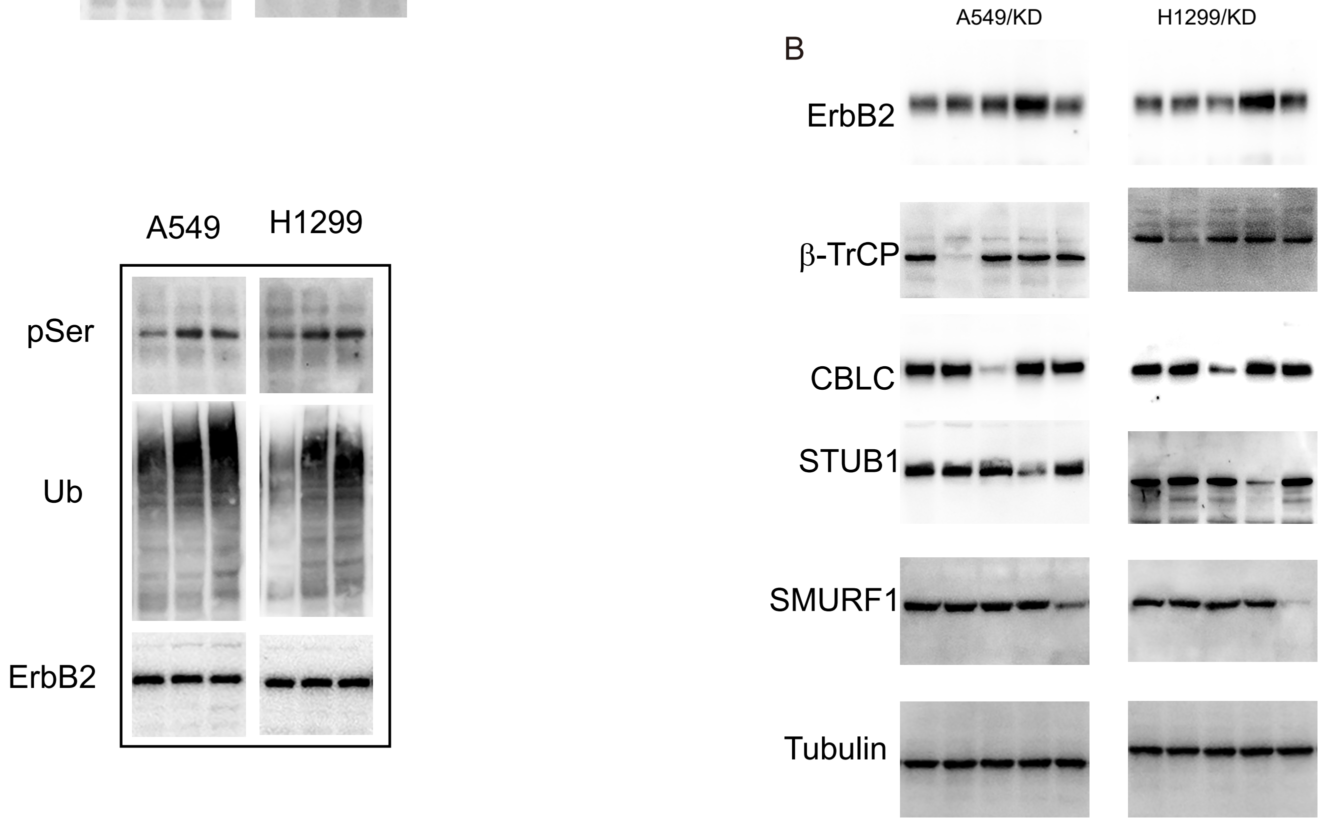

Figure 5

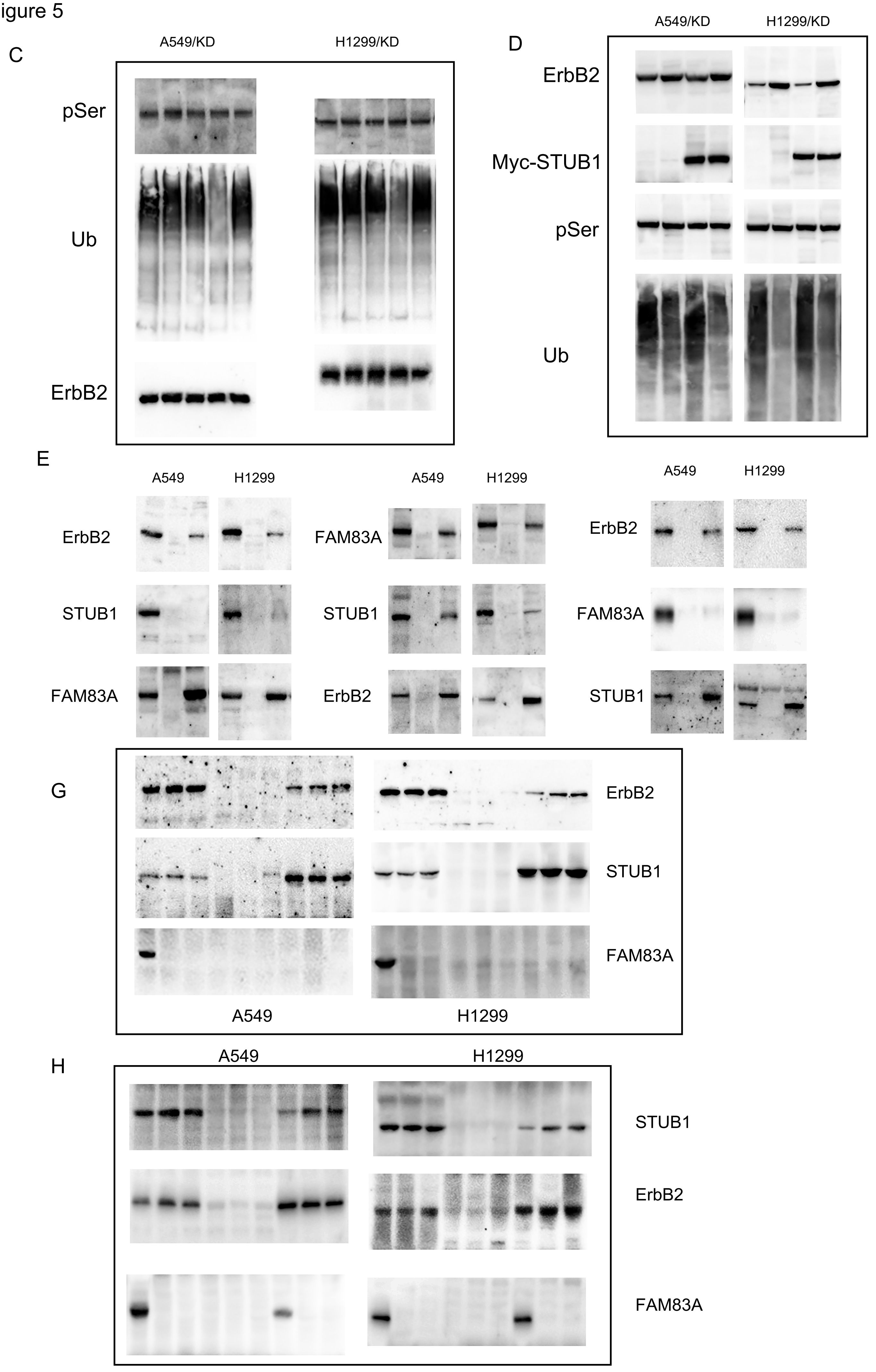
C
A549/KD
H1299/KD
pSer
Ub
ErbB2
D
A549/KD
H1299/KD
ErbB2
Myc-STUB1
pSer
Ub
E
A549
H1299
ErbB2
STUB1
FAM83A
A549
H1299
FAM83A
STUB1
ErbB2
A549
H1299
ErbB2
FAM83A
STUB1
G
ErbB2
STUB1
FAM83A
A549
H1299
H
A549
H1299
STUB1
ErbB2
FAM83A

Figure 6

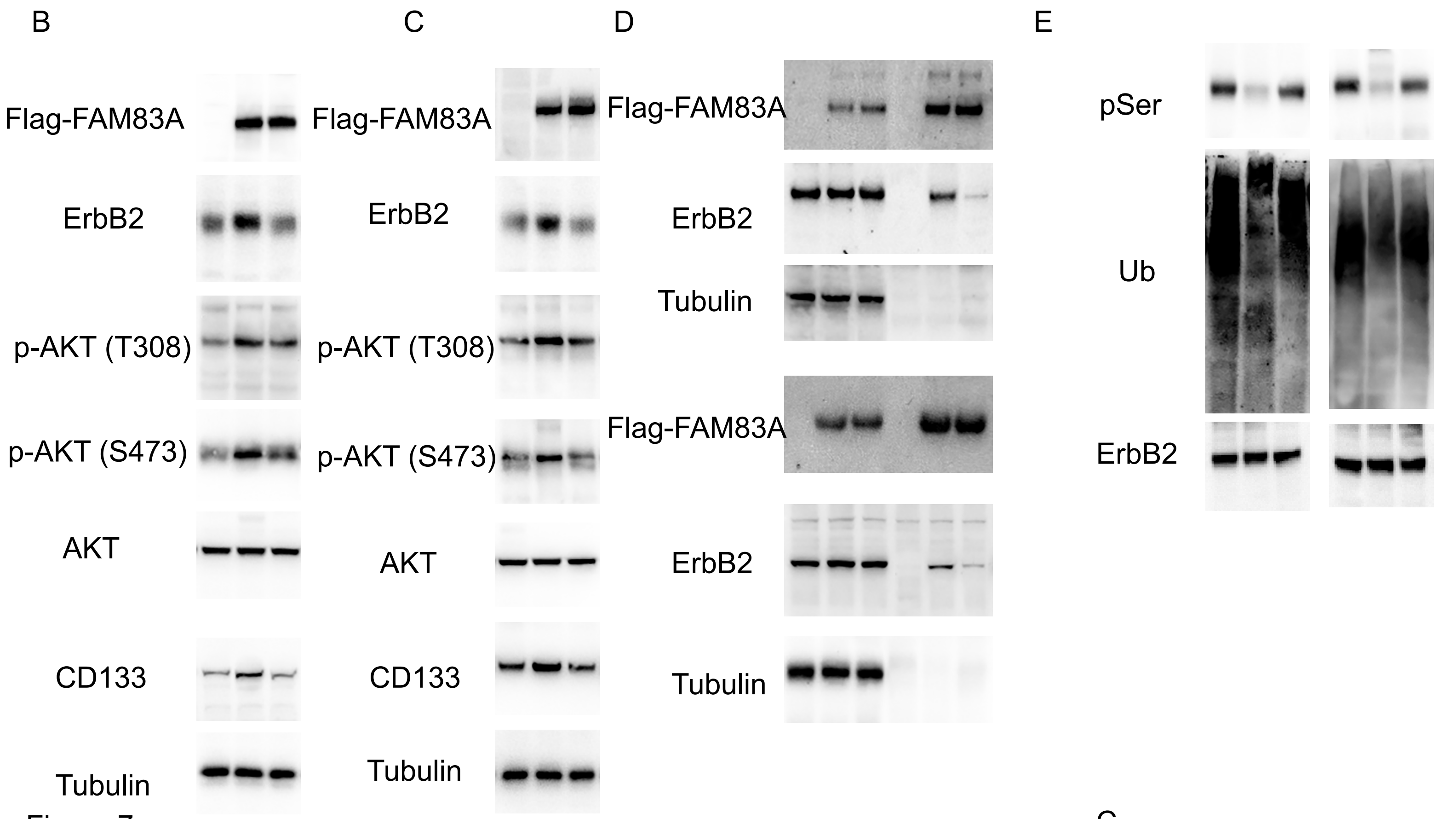

Figure 7

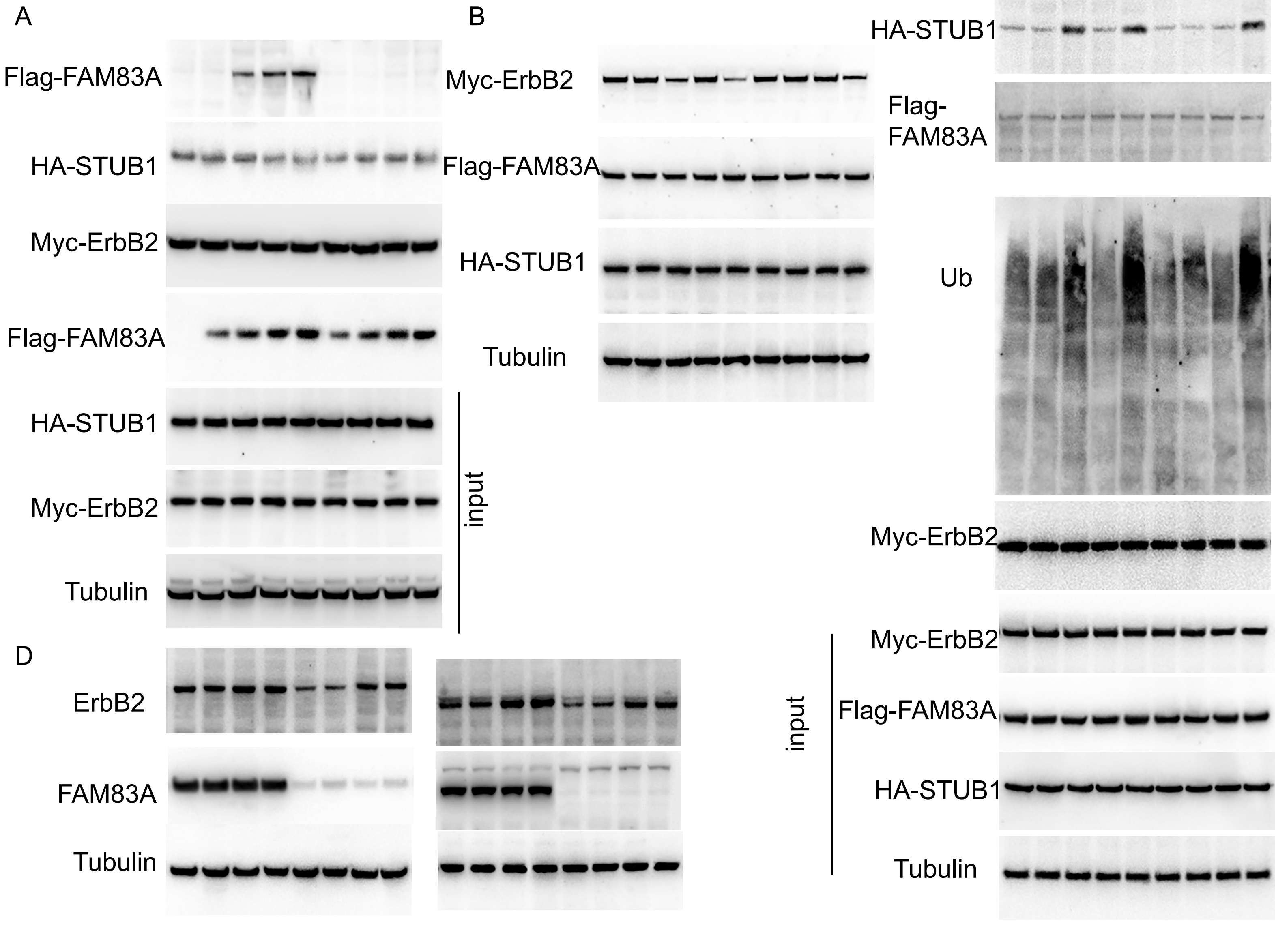

Figure 7

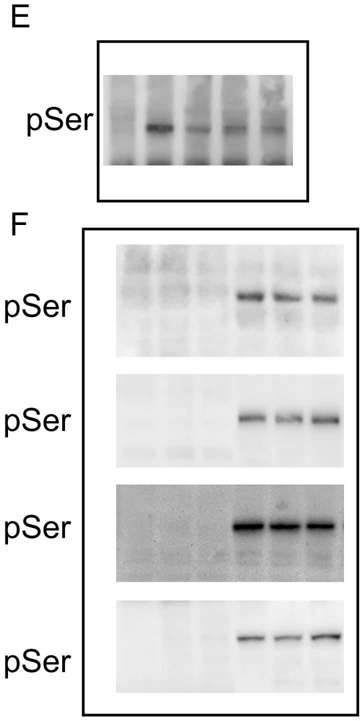

Figure 8

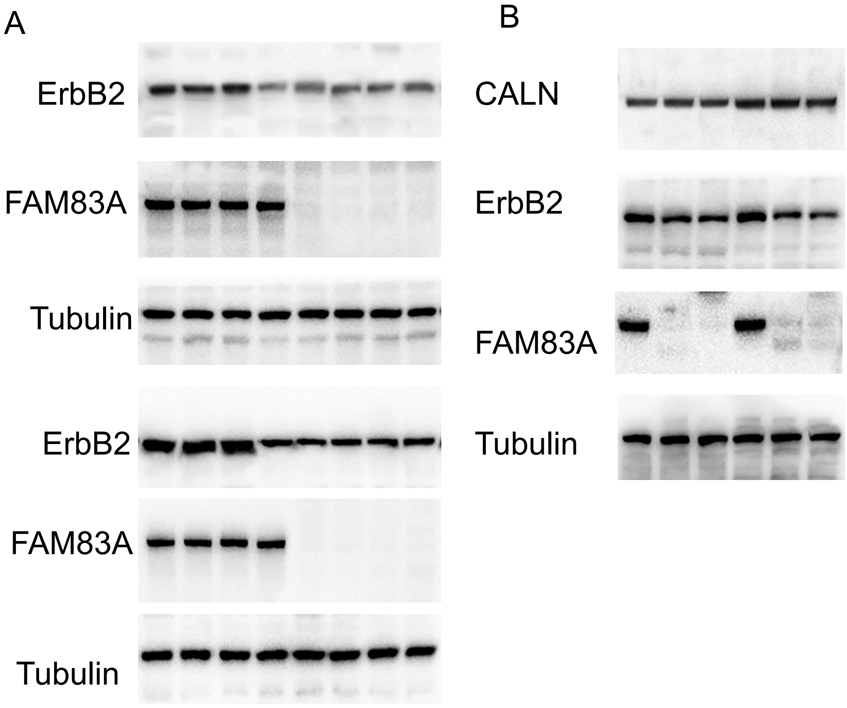

Figure 8

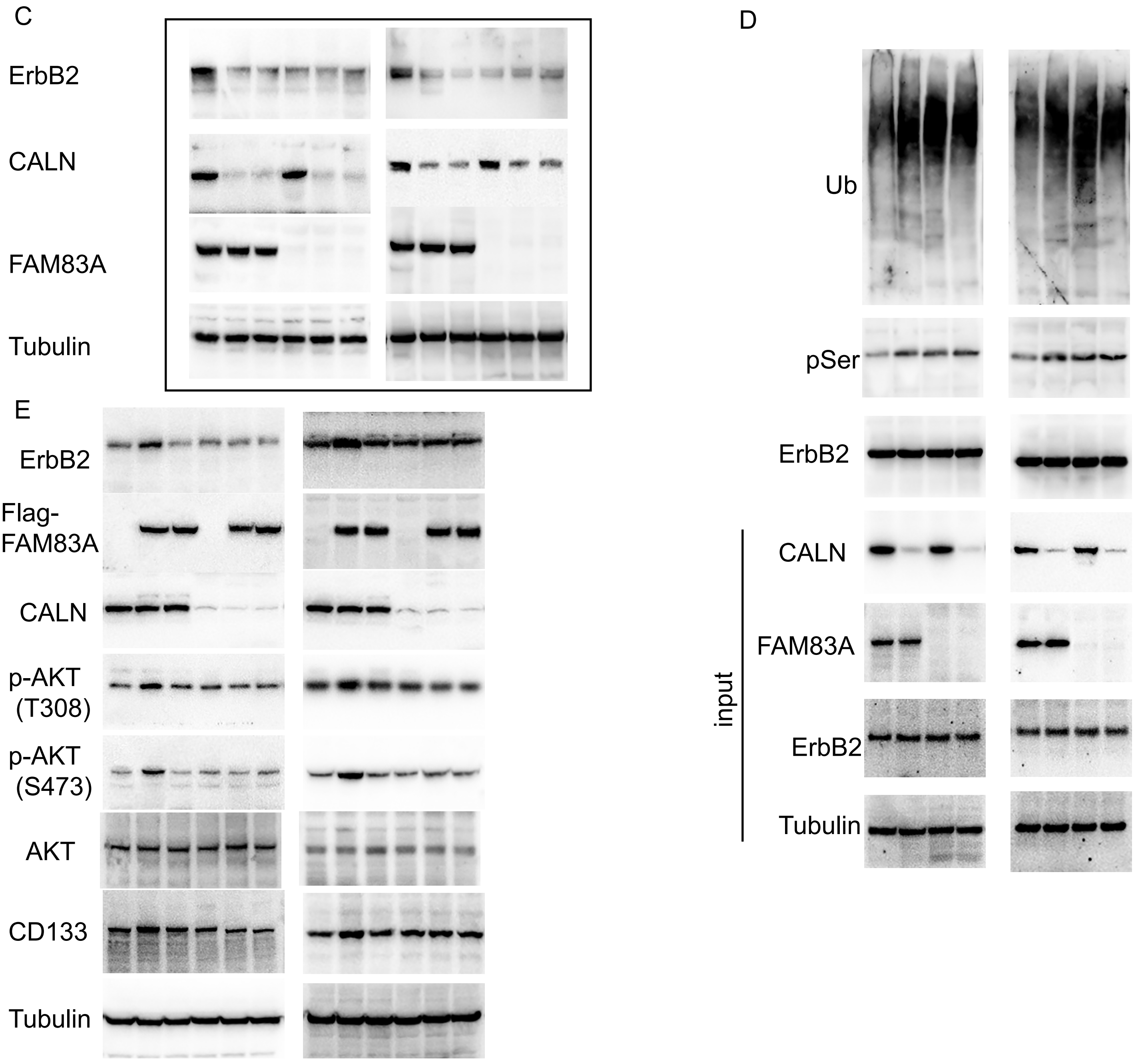

Supplement: Supplementary file 1 — Related Manuscript File-Raw data for WB [file 41419_2024_6853_MOESM1_ESM.pdf]
