# Supplementary material for: Promotion of stem cell-like phenotype of lung adenocarcinoma by FAM83A via stabilization of ErbB2
Source: Cell Death Dis. 2024 Jun 28;15(6):460. doi: 10.1038/s41419-024-06853-w (PMC11213963; doi:10.1038/s41419-024-06853-w)
Supplement: Supplementary file 3 — Figure S1 figure legends [file 41419_2024_6853_MOESM3_ESM.pdf]

Figure S1. A. RT-PCR of ErbB2 mRNA in control and FAM83A knockdown A549 and H1299 cells. B. Scatter plots showing the positive correlation between FAM83A and ErbB2 expression in the TCGA human LUAD datase, analyzed using Pearson’ s correlation test.
